# The U-Box E3 Ubiquitin Ligase TUD1 Functions with a Heterotrimeric G α Subunit to Regulate Brassinosteroid-Mediated Growth in Rice

**DOI:** 10.1371/journal.pgen.1003391

**Published:** 2013-03-14

**Authors:** Xingming Hu, Qian Qian, Ting Xu, Yu'e Zhang, Guojun Dong, Ting Gao, Qi Xie, Yongbiao Xue

**Affiliations:** 1 State Key Laboratory of Molecular Developmental Biology, Chinese Academy of Sciences and National Center for Plant Gene Research, Beijing, China; 2 National Rice Research Institute, Chinese Academy of Agricultural Sciences, Hangzhou, China; 3 Graduate University, Chinese Academy of Sciences, Beijing, China; 4 State Key Laboratory of Plant Genomics, Institute of Genetics and Developmental Biology, Chinese Academy of Sciences and National Center for Plant Gene Research, Beijing, China; University of Michigan, United States of America

## Abstract

Heterotrimeric G proteins are an important group of signaling molecules found in eukaryotes. They function with G-protein-coupled-receptors (GPCRs) to transduce various signals such as steroid hormones in animals. Nevertheless, their functions in plants are not well-defined. Previous studies suggested that the heterotrimeric G protein α subunit known as D1/RGA1 in rice is involved in a phytohormone gibberellin-mediated signaling pathway. Evidence also implicates D1 in the action of a second phytohormone Brassinosteroid (BR) and its pathway. However, it is unclear how D1 functions in this pathway, because so far no partner has been identified to act with D1. In this study, we report a *D1* genetic interactor *Taihu Dwarf1* (*TUD1*) that encodes a functional U-box E3 ubiquitin ligase. Genetic, phenotypic, and physiological analyses have shown that *tud1* is epistatic to *d1* and is less sensitive to BR treatment. Histological observations showed that the dwarf phenotype of *tud1* is mainly due to decreased cell proliferation and disorganized cell files in aerial organs. Furthermore, we found that D1 directly interacts with TUD1. Taken together, these results demonstrate that D1 and TUD1 act together to mediate a BR-signaling pathway. This supports the idea that a D1-mediated BR signaling pathway occurs in rice to affect plant growth and development.

## Author Summary

Rice is an important and staple grain food. Understanding the molecular basis of rice growth and development is crucial to safeguarding our food security. Hormone signaling pathways are key regulators of plant growth and development. Heterotrimeric G-protein complexes serve as signal transducers between cell surface receptors and downstream effectors. In plants, the repertoire of G-protein signaling elements is smaller than in mammals, but there are many examples of G protein components mediating important physiological responses. In rice, the heterotrimeric G protein α subunit known as D1/RGA1 appears to be involved in gibberellin and brassinosteroid (BR) responses, but it remains unclear how D1 functions in these responses. Here we discovered a *D1* genetic interactor *Taihu Dwarf1* (*TUD1*) that encodes a U-box E3 ubiquitin ligase. Such ligases are important regulators of cell functions. Genetic and molecular analyses revealed that D1 and TUD1 genetically and physically interact with each other and function together to regulate BR-mediated growth in rice. Furthermore, similar genes to *TUD1* are found in both Arabidopsis and other cereal species, such as *Sorghum bicolor* and *Zea mays*. G-protein signaling mediated by TUD1, therefore, may be conserved across flowering plants. Such key signaling molecules may provide a target to increase plant productivity by modulating the strengths of signals controlling growth in different tissues such as the seed grain.

## Introduction

Brassinosteroids (BRs) are a class of polyhydroxylated sterol derivatives, structurally similar to animal steroids, which appear to be ubiquitously distributed throughout the plant kingdom [1]. As a group of growth-promoting steroid hormones, they play pivotal roles in promoting cell expansion and division, regulating senescence, male fertility, fruit ripening and modulating plant responses to various environmental signals [1], [2]. Extensive studies in Arabidopsis have identified a nearly complete BR signaling pathway starting with BRI1 (BRASSINOSTEROID INSENSITIVE 1) as the cell membrane receptor which perceives and binds to BRs [3], initiates a phosphorylation-mediated cascade involving BSK1 (BR SIGNALING KINASE 1), BSU1 (BRI1-SUPPRESSOR1), BIN2 (BRASSINOSTEROID INSENSITIVE2), and PP2A (Protein phosphates 2A) and which subsequently transduces the extracellular steroid signal to the transcription factor BZR1 (BRASSINAZOLE RESISTANT 1) [1], [2], [4], [5]. In rice (*O.sativa*), the BRI1-mediated BR pathway appears to be conserved with Arabidopsis as several important components of this signaling pathway such as OsBRI1, OsBZR1 and 14-3-3 have the same function as their Arabidopsis orthologs [6], [7], [8]. Further, numerous BR-insensitive mutants in tomato, barley and pea have been identified as mutations in BRI1 orthologs [9], [10], [11], [12], indicating that the BRI1 pathway is conserved in flowering plants [7], [8].However, a major question for both Arabidopsis and rice remains; how do G proteins fit into this cascade? Several previous reports indicate that the canonical heterotrimeric Gα of rice and Arabidopsis are involved in a BR response but are apparently not related to the BRI pathway [13], [14], [15].

Heterotrimeric G proteins consist of three subunits, Gα, Gβ and Gγ, which play essential roles in various biological processes in many eukaryotes [16]. Once a ligand binds to a GPCR, the GPCR undergoes a conformational change which activates the G proteins by promoting the exchange of GDP/GTP associated with the Gα subunit, leading to its dissociation from Gβ/Gγ. Subsequently, Gα acts in its cascade while Gβ/Gγ regulate their own downstream effectors [17], [18]. In contrast to mammals that possess 16 Gα, 5 Gβ and 14 Gγ genes [19], Arabidopsis has 1 Gα, 1 Gβ and 3 Gγ (1 atypical) genes, while rice has 1 Gα, 1 Gβ and 5 Gγ (3 atypical) genes [18], [19], [20]. Despite this limited number of plant G protein members, their functions are diverse and have multiple roles in various hormone responses [16], [21]. In particular, a loss-of-function mutant in the rice Gα gene D1/RGA1, displays dwarfism, erect leaves, compact panicle and small round seeds. The *d1* mutant was originally identified as a gibberellic acid signaling mutant and exhibited reduced growth and a highly hypersensitive response to infection by a fungus [22], [23], suggesting that D1 is involved in both GA signaling pathway and disease resistance. However, several recent studies have shown that the phenotypic characteristic of the *d1* mutants are more similar to that of BR-deficient mutants, displaying shortened second internodes, erect leaves, constitutive photomorphogenic growth in darkness and decreased sensitivity to the brassinosteroid 24-epibrassinolide (24-eBL) [13]. Importantly, double mutants obtained from crossing *d1-7* and *d61-1* (an OsBRI1 allelic mutant) showed no epistasis in many organs except in seed length and seed weight [14], [24]. In addition, the expression patterns of several BR biosynthetic genes were not altered by brassinosteroid in *d1* mutants. These results indicated that there may exist a BR signaling pathway in rice which involves Gα, but which is different from the canonical BRI1 pathway [25]. This idea is in agreement with the results for the Arabidopsis Gα gene (*GPA1*). The mutant *gpa1* shows less sensitivity to 24-eBL and double mutants between Gα-deficient mutants and BR-deficient mutants had additive effects in many organs and tissues [15]. Thus, it is important to understand this potentially novel Gα-mediated BR pathway and to show how it controls BR-mediated growth responses.

Recent studies have shown that the ubiquitin-proteasome system (UPS) is an integral part of auxin, GA, jasmonic acid (JA), ethylene and abscisic acid signaling or biosynthetic pathways [26]. UPS is regarded as one of the most prominent mechanisms which regulates protein degradation to modulate protein levels in plants to efficiently alter their proteomes and so ensure proper developmental responses and environmental adaptations [27]. Ubiquitin is a 76 amino acids polypeptide that is covalently attached to a protein target through an enzymatic cascade comprising a ubiquitin-activating enzyme (E1), a ubiquitin-conjugating enzyme (E2), and a ubiquitin ligase (E3). The E3s are key factors that define substrate specificity. In plants four main types of E3s have been classified according to their mechanisms of action and subunit composition: HECT, RING, U-box and Cullin-RING ligases (CRLs) [26]. U-box E3 ligases are grouped based on a conserved 70 amino acid motif, that lacks characteristic zinc-chelating cysteine and histidine residues, and so uses intramolecular interactions to maintain the U-box scaffold [28], [29]. Yeast and humans have 2 and 21 U-box genes, respectively. In contrast, Arabidopsis and rice have 64 and 77 U-box genes, respectively [30], [31]. The expansion of the plant U-box gene family suggests that they are key in controlling diverse cellular processes, with possibly many being specific to plants. The biological functions of over 25 U-box E3s have been reported, involving hormone responses, biotic stress, abiotic stress and self-incompatibility [32], [33], [34], [35]. Here we characterize a new U-box E3 function. We have identified the U-box E3 ligase gene, *TAIHU DWARF1* (*TUD1*) and shown its genetic interactions with the rice Gα subunit D1. TUD1 is a functional E3 ligase and acts as a BR signaling activator. Furthermore, TUD1 and D1 physically interact and together define a D1-mediated BR signaling pathway which may parallel or partly overlap with the canonical BRI1 pathway.

## Results

### Identification of a genetic interactor with *D1*

**Figure 1:**
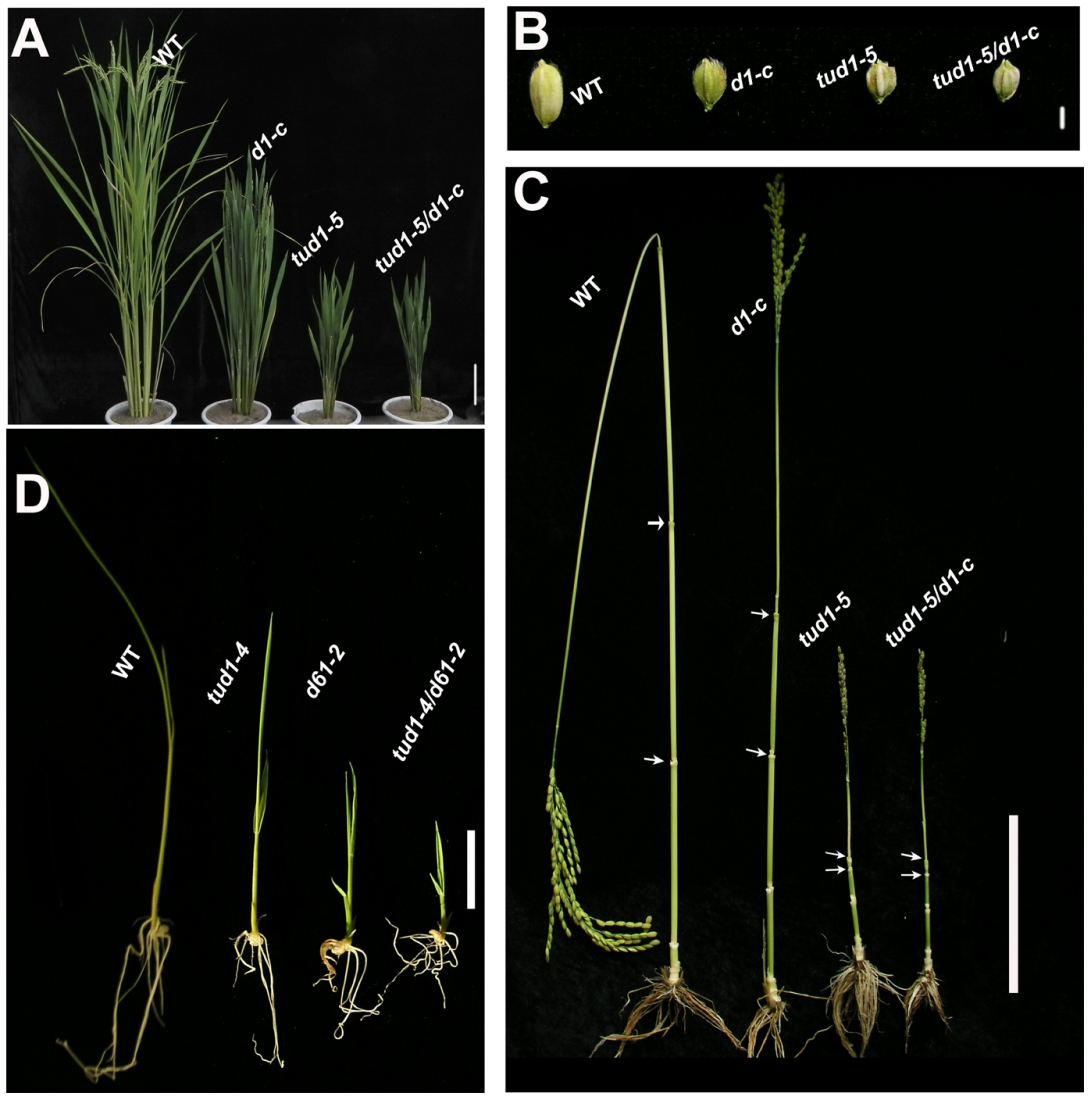
*tud1* is epistatic to *d1*, but additive to *d61*. (A) Adult plant morphology of wild type (WT) (Nipponbare), *d1-c*, *tud1-5* and *d1-c*/*tud1*. Both *tud1-5* and *d1-c* mutants were derived from Nipponbare. The plant height of *tud1* is similar to *d1-c*/*tud1*, showing that *tud1* is epistatic to *d1* in plant height. Bar: 10 cm. (B) The grain morphology of wild type (WT), *d1-c*, *tud1-5* and *d1-c*/*tud1* mutants. The vertical grain lengths of *tud1-5*, *d1-c* and *d1-c*/*tud1* were shortened compared to wild type. The vertical grain length of *tud1-5* is similar to *d1-c*/*tud1*, showing that *tud1* is also epistatic to *d1* in grain length. (C) The patterns of internode elongation in wild type (WT), *d1-c*, *tud1-5* and *d1-c*/*tud1*. *d1-c* is characterized by a dn-type dwarfism, but *tud1* and *d1-c*/*tud1* are of dm-type dwarfism and their second internode elongations are specifically inhibited. Arrows indicate start and end of the second internode. Bar: 10 cm. (D) Plant heights for wild type (WT), *tud1-4*, *d61-2* and tud1/d61, showing that these mutants are additive in the reduction of plant height. Bar: 2 cm.

To dissect additional components involved in *D1*-dependent BR responses [13], [14], we took a genetic approach to identify mutants similar to *d1*. Among 250 reduced plant height mutants of rice, a dwarf mutant similar to *d1* was identified and subsequently shown to be non-allelic to *d1*. We named this dwarf mutant *taihu dwarf1* (*tud1*). Five allelic mutants (*tud1-1* to -*5*) were further identified from our dwarf mutant collection. To examine the genetic relationship of *d1* and *tud1*, we crossed a weak *d1* allele with a strong *tud1* allele (*tud1*-*5*) with dm-type dwarfism (see below) in the same background of Nipponbare [36]. The phenotype of the F_1_ was normal, suggesting that they were non-allelic. The phenotype of the double mutant in the F_2_ was similar to *tud1*-*5* with a specific reduction of the second internode length, erect leaves and shortened grain lengths (Figure 1A–1C), indicating that *tud1*-*5* was epistatic to *d1-c* (Table S1). In addition, *tud1-4* and *d61-2* (an *OsBRI1* allelic mutant) were crossed and the double mutant showed that *tud1* and *d61* had an additive effect on rice growth and development (9∶3∶3∶1,χ^2^:0.677, p>0.05 Figure 1D). These results showed that *TUD1* acts in the same genetic pathway as *D1* but different from that involving the rice BRI1 ortholog *D61*.

### *tud1* is a pleiotropic dwarf mutant

**Figure 2:**
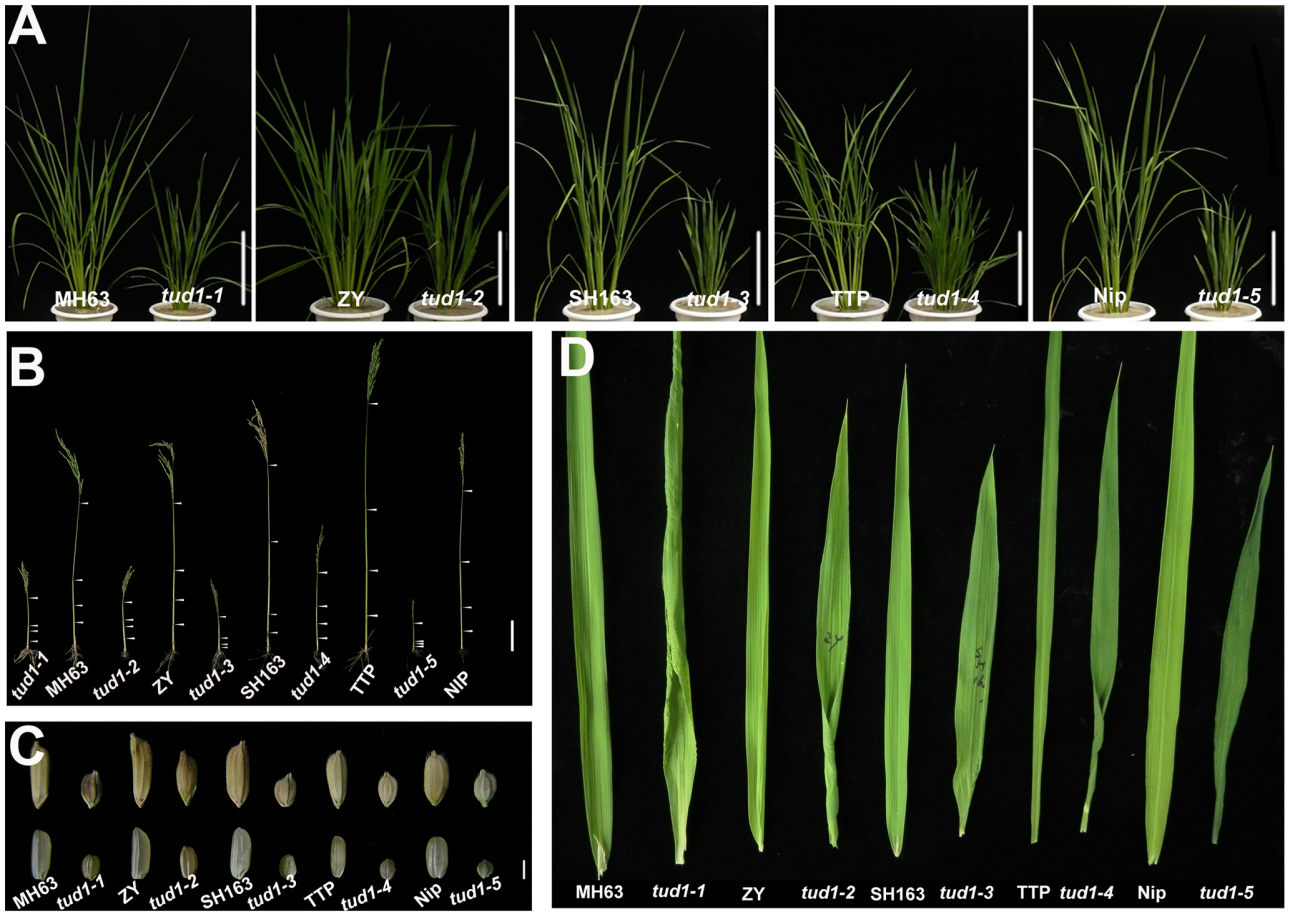
*tud1* is a pleiotropic dwarf mutant. (A) Adult plant morphology. Comparison of five allelic mutants of *tud1* (*tud1-1* to *-5*) with their corresponding wild rice varieties MH63, ZY, SH163, TTP and Nip, respectively. Bar: 10 cm. (B) Comparison of culm (stem) elongation of the *tud1* mutant alleles with their corresponding wild types. According to Takeda (1977), *tud1-1*, *tud1-2* and *tud1-5* showed dm-type dwarfism, and *tud1-3* and *tud1-4* showed dn type. Arrowheads indicate the positions of nodes. Bar: 10 cm. (C) Grain morphology. All of the mutants have shortened grains. The upper panel and the lower panel represent the unhulled and hulled seeds, respectively. Bar: 2 mm. (D) Leaf morphology. The leaf blades of *tud1* mutants all are dark-green and rugose.

To examine the type of dwarfism of *tud1*, we compared the gross morphology of 9-week-old wild-type and *tud1* plants (Figure 2A). The plant heights of *tud1* mutants were significantly shorter than their corresponding wild type, and *tud1-5* showed a severe dwarf phenotype. The internode elongation was inhibited in all of the mutants. Lengths of the individual internodes of plants were measured and expressed as a relative value (Figure 2B). Among them, *tud1-1*, *tud1-2*, and *tud1-5* showed a specific internodal inhibition; the second internode was severely shortened relative to other internodes. This pattern was typically classified as a dm-type elongation pattern [36]. In *tud1-3* and *tud1-4*, the lengths of each internode were almost uniformly shortened, resulting in an elongation pattern similar to that of wild type. According to Takeda [36], *tud1-3* and *tud1-4* showed dn-type dwarfism (Figure S1). To our knowledge, rice mutants showing different patterns of inhibition of internode elongation were only reported in BR-insensitive mutants of *d61* and *d1* [6], [14], suggesting that *tud1* is also involved in a BR pathway. In addition, either unhulled or hulled seeds were specifically shortened in the vertical direction in *tud1* mutants (Figure 2C). Compared with their corresponding wild type, the grain lengths of *tud1* mutants were reduced by 30 to 44%. Their leaves were also shortened, erect and dark-green, similar to *d1* (24), but their severe rugose (curled) nature appeared to be different from *d1* (Figure 2D).

**Figure 3:**
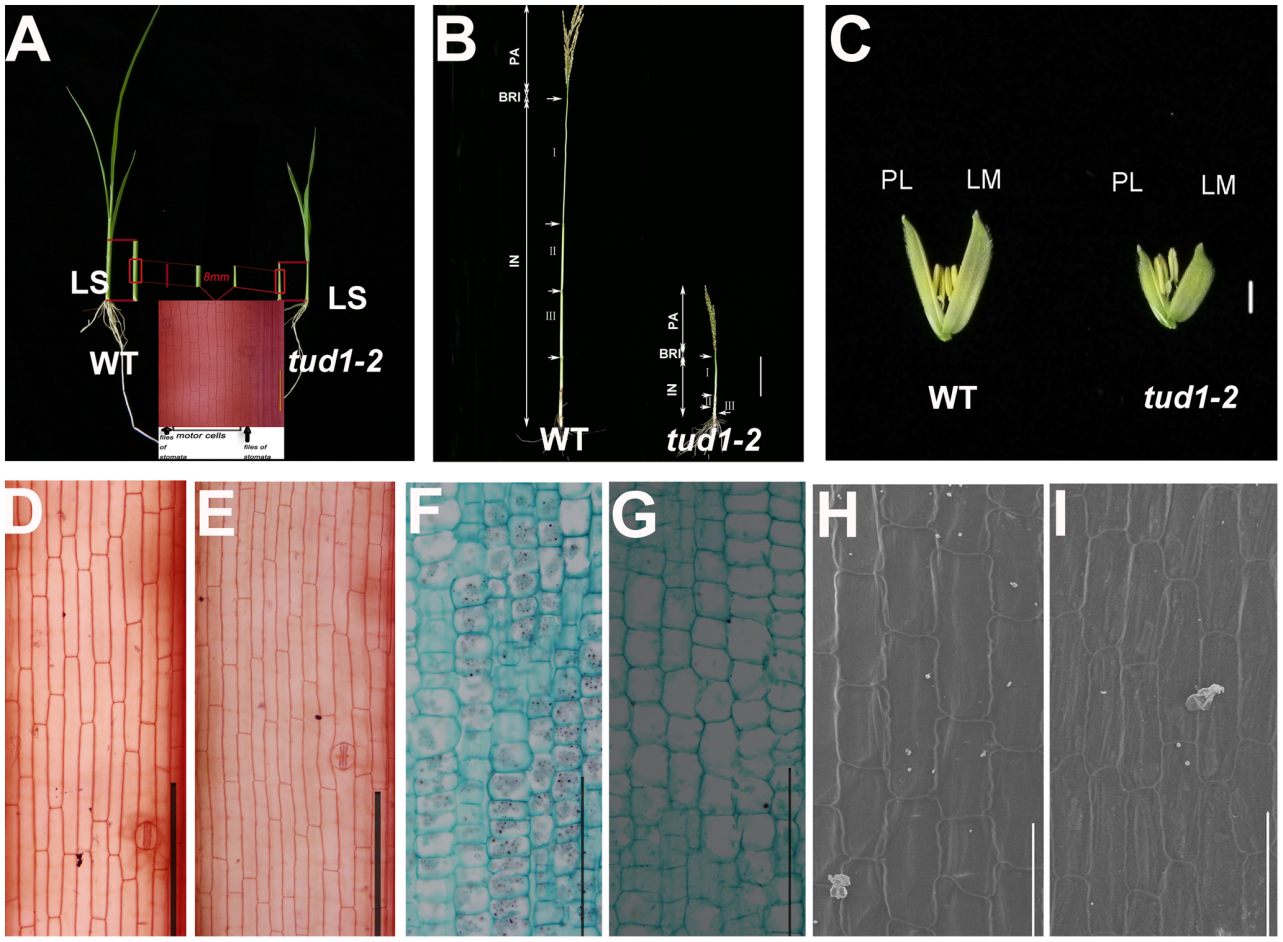
Cell numbers are reduced in aerial organs of *tud1-2*. (A) Aerial parts of three-week old wild type (WT) (upper-left) and *tud1-2* (upper-right). Schematic illustration of the third leaf sheaths is shown in middle bottom. Central parts of the leaf sheaths were cut off and the lengths of motor cells between files of stomata were measured. (B) Panicles and internodes of wild type (WT) and *tud1-2*. PA, panicle; BRI, basic rachis internode; IN, internode. Internode I, II and III are the first, second and third internodes, respectively. Bar: 10 cm. (C) Lemma and palea of WT and *tud1-2*. LM: Lemma; PL: Palea. Bar: 2 mm. (D,E) Longitudinal sections of the third leaf sheath from one-month old seedlings of wild type (WT) (D) and *tud1-2* (E). Bar: 200 µm. (F,G) Longitudinal sections of the third internode from one-month old seedlings of wild type (WT) (F) and *tud1-2* (G). Bar: 200 µm. (H,I) Inner epidermal cells of lemma from one-month old seedlings of wild type (WT) (H) and *tud1-2*(I) observed by SEM. Bar: 100 µm.

To determine whether dwarfism in *tud1* was due to cell division, cell elongation or both, we measured the cell length and number in the third leaf sheath, the third internode and the lemma of *tud1-2* and its wild type (Figure 3A–3C). Overall we found that the total number of cells, in all given organs, was reduced in the mutant compared to wild type (Table S2). The length of the third leaf sheath in *tud1-2* was decreased by 57% compared to wild type, while the average cell length was similar. Thus, the estimated cell number in the third leaf sheath was reduced by about 43% compared with the wild type, indicating that the shortened leaf sheath in *tud1-2* was due to a reduction in cell number rather than cell length (Figure 3D, 3E). Similarly, the calculated cell number of the third internode and the lemma in *tud1-2* was reduced by about 67% and 36% respectively (Figure 3F–3H). These results indicated that *tud1* has a significantly reduced cell number in these aerial plant organs.

We analyzed more closely the changes in the second internode and adaxial leaf surfaces. In the wild type, cells in the second internode were elongated and well-organized into longitudinal files, but in *tud1-2* and *tud1-1*, cell files were disorganized and not elongated (Figures S2, S3). In the wild type, the epidermal cells of leaf blade almost run parallel to the vertical vascular tissues, but in *tud1-2*, all of the vertical tissues were waved and the leaf epidermal cells had a disorganized arrangement (Figure S4). However, it was clear that the extent of the overall disorganization of leaf epidermal cells was less severely affected in tud1-2 than in the second internodal cells. These results showed that the deficiency of cell division and the arrangement of poorly organized cell files leads to dwarfism in *tud1* plants.

### *TUD1* is involved in BR responses but not in gibberelin or cytokinin responses

**Figure 4:**
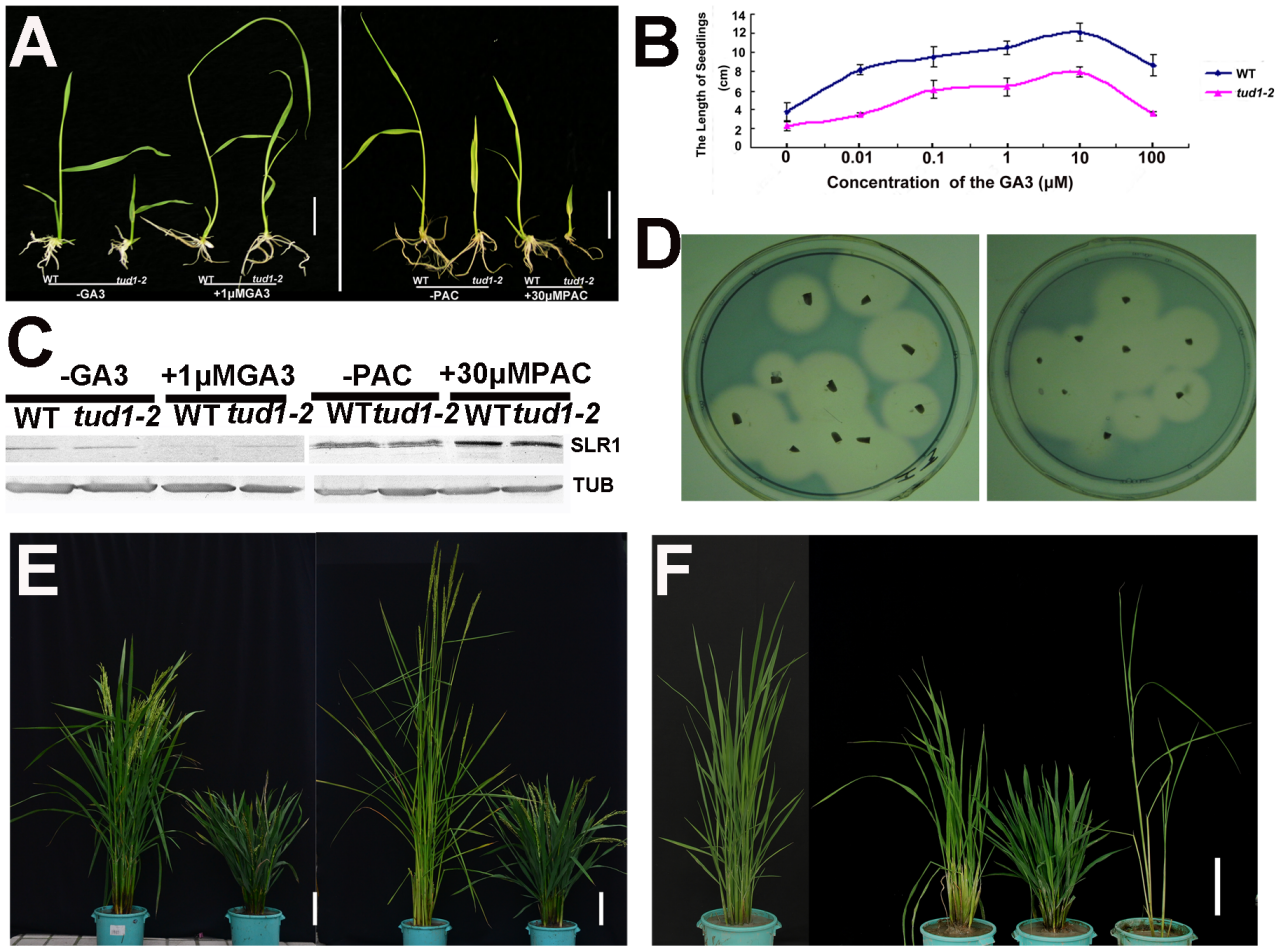
*tud1-2* is unlikely a GA-related mutant. (A) Seeds of wild type (*TUD1*) and the dwarf mutant (*tud1-2*) were germinated on agar plates in the presence (+) or absence (−) of 1 µM GA_3_ (Left), or 30 µM of PAC (Right), and seedlings were examined 10 days after germination. (B) Seedling lengths (heights) in response to GA3 treatment in the wild type (blue) and *tud1-2* (pink). (C) Changes in SLR1 protein levels triggered by GA_3_ or PAC treatment. Young seedlings of the wild type (WT) and *tud1-2* were treated with (+) 1 µM GA_3_, 30 µM PAC or control solution (−), as described in Figure 4A and 4B, and their extracts subjected to western blot analysis by anti-Tubulin and anti-SLR1 antibodies, respectively. (D) A plate assay for α-amylase induction. Left: wild type (WT); Right: *tud1-2*. (E) Gross morphology of *tud1-2* and a new *eui1* mutant in the *tud1-2* background. From left to right; wild type (WT), *tud1-2*, *eui1-h,tud1-2/eui1* (right). Bar: 10 cm. (F) Gross morphology of *tud1-2*, *slr1* and *tud1-2/slr1* mutants. From left to right; wild type (WT), *tud1-2/slr1*, *tud1-2* and *slr1* (right). Bar: 15 cm.

Because of its dwarf phenotype, *d1* was initially classified as a gibberellin (GA)-insensitive mutant [22]. To examine whether *tud1* was a GA-deficient mutant, we performed GA and paclabutrazol (PAC, an inhibitor GA biosynthesis) application assays for *tud1-2* and wild type. Treatment of *tud1-2* plants with 1 µM GA_3_ or 30 µM PAC had effects similar to those seen in wild type (Figure 4A). Similarly, the effect of different concentration of GA3 on increase of seedling plant height was also similar between the wild type and *tud1-2* (Figure 4B), indicating that *tud1-2* plants have a normal sensitivity to GA. To further confirm this, we performed an immunoblot analysis of SLENDER1 (SLR1) protein, which is a repressor in the GA signaling pathway [37], [38], and found that SLR1 protein levels remained similar in *tud1-2* and wild type plants treated by either GA_3_ or PAC (Figure 4C). Meanwhile, an α-amylase activity assay (diagnostic for GA responses) also showed that wild type and *tud1-2* responded similarly to GA (Figure 4D). Additionally, a new *eui1-d* (*elongation uppermost internode1-d*) mutant, which accumulated exceptionally large amount of biologically active GAs in the uppermost internode [39] in *tud1-2* background, was recovered and showed additive phenotypes (Figure 4E), indicating that *tud1* did not influence the GA biosynthesis. Furthermore, a *tud1-2*/*slr1-l* double mutant exhibited additive plant height phenotypes (Figure 4F). Taken together, these results showed that *tud1-2* is not a GA-deficient mutant.

To ascertain whether or not the *tud1* is a cytokinin (CK)-related mutant, the seeds of *tud1-2* and its wild type were germinated on agar plates with or without the cytokinin 6-benzyladenine (6-BA). The growth of the seminal (main) root of both wild type and *tud1-2* were inhibited and the degree of reduction in root length was similar in both. Also, the relative expression of three CK-related genes *OsIP4*, *OsRR1*, and *OsHP2* (Table S4) [40] were not significantly altered between wild type and *tud1-2* (Figure S5). Additionally, the degree of the alteration in the relative expression level of *OsIP4*, *OsRR1*, *OsHP2* were also found to be similar in wild type and *tud1-2* with treatment of 6-BA when compared to no treatment (Figure S6A, S6B, S6C). These results showed that *tud1-2* is not a CK-deficient mutant and its phenotype is not directly associated with CK.

**Figure 5:**
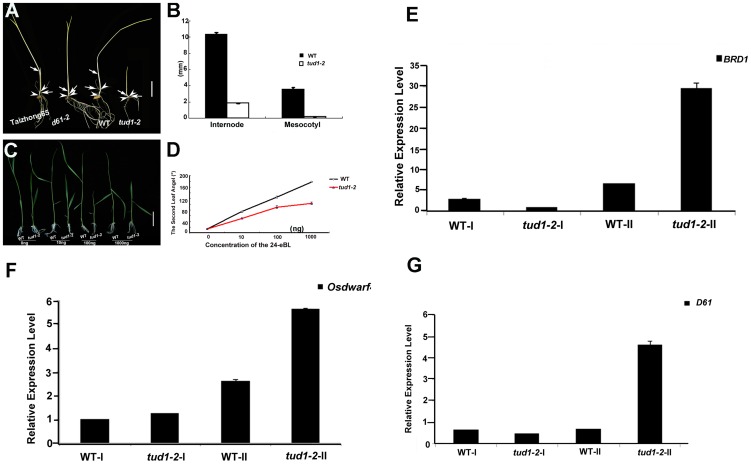
*tud1-2* is a BR insensitive mutant. (A,B) Photomorphogenic phenotypes of *tud1* grown in the dark. Plants of Taizhong 65 (WT), *d61-2* (BR-insensitive mutant), wild type (WT) and *tud1-2* were grown in complete darkness (A). Arrows indicate nodes and arrowheads. Bar: 2 cm. The lengths of internodes and mesocotyls were measured after ten days growth. Data presented are the means of results from five plants (B). Error Bars = SD. (C,D) Effects of 24-eBL on the degree of inclination of the leaf lamina in wild type (WT) and *tud1* plants. Typical responses of the second leaf lamina joint from wild type and *tud1-2* plants to 24-eBL at 0 ng, 10 ng, 100 ng or 1000 ng. Bar: 2 cm (E). The dose responses to 24-eBL, for the bending angle, for WT and *tud1-2* (F). Data presented are the means of results from a total of five plants. Error Bars = SD. (E,F,G) Relative expression levels of *BRD1* (E), *OsDWARF4* (F) and *D61* (G) in different internodes. WT-I and *tud1-2*-I represent the uppermost internode, WT-II and *tud1-2*-II represent the second elongated internode, respectively (from top to bottom).

To examine whether *tud1* was involved in BR responses, we first performed a mesocotyl elongation experiment in the dark. The *tud1-2* mutant showed a typical deetiolated phenotype, similar to the BR-insensitive mutant *d61-2* [6] (Figure 5A and 5B). Next, seeds of wild type and *tud1-2* mutant plants were germinated on agar plates containing different concentrates of 24-eBL and examined for the length of the seminal root after one week. The growth of the seminal root of wild type was inhibited by 24-eBL in a dose-dependent manner, but the *tud1-2* mutant showed a significantly reduced response to 24-eBL (Figure S7A and S7B). The growth of the seminal root was inhibited 43% in the wild type, but only 27% in the *tud1-2* mutant in the presence of 10^−6^ M 24-eBL (Figure S7B), suggesting that *tud1* is much less sensitive to exogenous BL than the wild type. Furthermore, a lamina joint test was performed using *tud1-2* and wild type [41]. In wild type, the lamina joint bending was increased in a dose-dependent manner, from 14.2° to 178.5° with 0 to 1000 ng 24-eBL (Figure 5C and 5D). In *tud1-2* plants, the degree of bending of the leaf lamina also increased with increased concentrations of 24-eBL, but the effect of the bent angles was much smaller than that of wild type under the same condition (Figure 5C and 5D). We compared the effects of 24-eBL on the size of cells in the lamina regions of the wild type and *tud1-2*. Under no treatment of 24-eBL, the adaxial cell number in the lamina regions of the wild type was similar to that in the *tud1-2*. However, in the presence of 24-eBL, the adaxial cells in this region of wild type were greatly expanded while the cells in *tud1-2* were only expanded slightly (Figure S8). These results indicated that the cells of lamina region in *tud1-2* were less insensitive to 24-eBL than that in the wild type. Therefore, these results show that *tud1* is a BR insensitive mutant.

### Negative feedback regulation of BR-related genes in the second internode and seedling leaf blade in the *tud1-2* mutant

The results of histological analysis above showed that the main cause of dwarfism in the second internode and leaf blade appeared to be different from that in most of the other aerial organs of *tud1-2*. To understand what is the molecular mechanism of retardation in these two type organs, we checked the relative expression levels of several BR-related genes as *tud1-2* is affected in BR responses. We analyzed the uppermost (first) and second internode of *tud1-2* and its wild type, and the seedling leaf blade of wild type, *d1-c*, *tud1-5* and *d1-c/tud1-5* with and without treatment of 24-eBL. The q-RT-PCR results showed that the amount of *BRD1*, *OsDWARF4* and *D61* [6], [42], [43], [44] expression differed significantly between the uppermost internode and the second internode; expression of all BR-related genes was stronger in the second internode of *tud1-2* than that in the uppermost internode (Figure 5E, 5F, 5G). Similarly, the q-RT-PCR results also indicated that relative expression level of *D61* was higher in the seedling leaf blade of the *d1-c/tud1-5* double mutant compared with its parents (Figure S9). The expression level of *D61* was also found to be higher in the second internode and seedling leaf blade in *tud1-2*. These results suggested that mutation in TUD1 impairs the OsBRI1-mediated BR pathway signal transduction. This affect has consequences with altered feedback regulation of the expression of *BRD1* and *OsDWARF4* occurring in the second internode of *tud1-2* mutant.

### *TUD1* encodes a functional U-box E3 ligase

**Figure 6:**
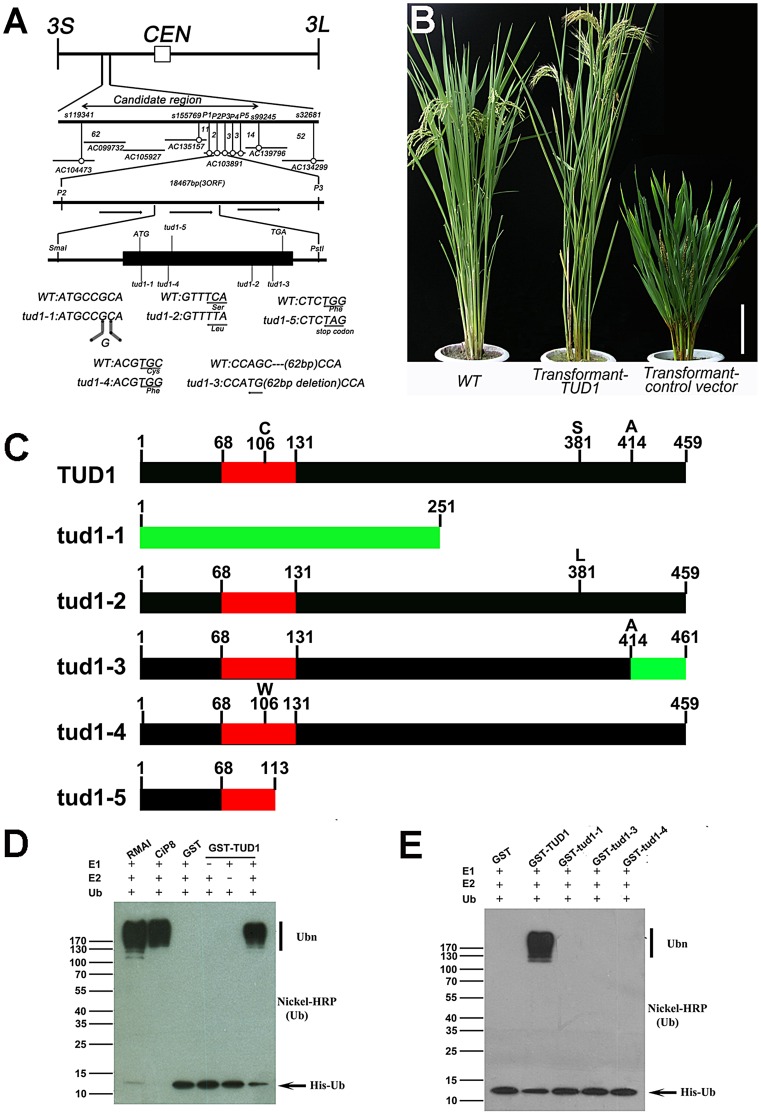
TUD1 is a functional E3 ligase. (A) High resolution linkage and physical map of *tud1* locus. Horizontal lines represent chromosome 3 and vertical bars the molecular markers. The numbers of recombinant plants are indicated between the markers. The physical map of the *tud1* locus was constructed using 7 BAC clones and the candidate region of the *tud1* mutation was found between markers P_2_ and P_3_. The genomic structure of the *TUD1* gene and the positions of mutations in *tud1* alleles are also shown. The black box indicates the single exon. The mutated DNA sequences of *tud1* alleles are shown in the bottom. The *tud1-1* has one-base insertion near the initiation codon. The *tud1-2*, *tud1-4* and *tud1-5* have different one-base substitutions in the coding sequence. The *tud1-3* has a one-base substitution and 62 bp base deletion in the exon. CEN:centromere, 3S: the short arm of chromosome 3, 3L: the long arm of chromosome 3. (B) Phenotypic complementation by introduction of the *TUD1* gene. Nipponbare was the parent for *tud1-2* and used as the wild-type plant. Left, the wild type (Nipponbare); center, transformant-*TUD1*; right, transformant-control vector. Bar: 10 cm. (C) Schematic structures of TUD1 protein and its mutant alleles. Black bars indicate TUD1 coding regions. Red bars depict U-box motifs. Green bars represent the aberrant truncated protein of tud1-1 and partial amino acids residues of tud1-3 due to a frameshift mutation. Numbers represent the amino acids of protein sequences. (D) Ubiquitination assays with GST-TUD1. GST-TUD1, GST, and positive controls of RMAI and CiP8 were assayed for E3 activity in the presence of E1 (from wheat), E2 (UBCh5b), and 6×His tag ubiquitin (Ub). The numbers at left denote the molecular mass in kilodaltons. Samples were resolved by 8% SDS-PAGE. The nickel-horseradish peroxidase was used to detect His tag ubiquitin. (E) Ubiquitination assays with GST-TUD1 and its mutant variants. GST-TUD1 and its mutant forms (GST-tud1-1, GST-tud1-3 and GST-tud1-4 fusion proteins) were assayed for E3 activity. The reaction conditions were the same as in (D).

To examine its molecular function, we isolated *TUD1* by map-based cloning. The *TUD1* locus was first mapped to the short arm of chromosome 3 between markers s1193411 and s32681 (Figure 6A, Table S5). *TUD1* was further localized to an 18.457 kb region containing three open reading frames (Figure 6A, Table S5). These reading frames were sequenced and the second one, annotated as a U-box protein (LOC_03g13010, Os03g0232600), was found to be mutated in all the five allelic mutants (Figure 6A). To further confirm the identity of *TUD1*, a DNA fragment of ∼6.4 kb in size including the entire sequence of the putative gene was introduced into *tud1-2* by *Agrobacterium*-mediated transformation [45]. Transformants with the *TUD1* gene-containing vector showed phenotypes similar to wild-type plants, while transformants with the control vector containing no target gene did not (Figure 6B). Thus, *tud1* was caused by a loss-of-function mutation in a U-box gene.

On the basis of public data (www.tigr.org and http://cdna01.dna.affrc.go.jp/cDNA/) and our results of 3′ RACE (3′ end rapid amplification cDNA ends), we found that *TUD1* is an intronless gene corresponding to an ORF(open reading frame) of 1380 bp, which is predicted to encode a protein containing 459 amino acids residues with a U-box motif near its N-terminus (Figure 6C). All five allelic mutants were shown to be mutated in the coding sequence (CDS), but at different locations. Despite their differences in genetic background, the *tud1-5*, *tud1-1* and *tud1-2* were easily grouped as strong alleles by their mutant phenotypes, showing the smallest leaf angles and most dwarfism in elongation of internodes when compared to wild type. In contrast, *tud1-3* and *tud1-4* exhibited mild mutant phenotypes, with respect to the elongation pattern of internodes. The degree of mutant phenotype strength appeared to be correlated to the severity of mutation in TUD1. *tud1-5* was identified as a null allele due to the change of 341^G^ into 341^A^ leading to a premature stop codon. *tud1-1* has a “G” inserted into the CDS of TUD1, near the initiator codon (ATG), and generates an aberrant truncated protein compared to TUD1 (Figure 6A, 6C). Although there is a single base substitution mutation both in *tud1-2* and *tud1-4*, the mutant phenotype of *tud1-2* was more severe than that of *tud1-4*, suggesting that the 381^S^ amino acid in TUD1 is very important (Figure 6A, 6C). *tud1-3* has a mild phenotype, likely due to a mild defective function in TUD1 with a 62 bp deletion in the CDS of TUD1, not entirely abolishing its function *in vivo*, but showing no *in vitro* ubiquitination activity (see below).

Blast searches revealed that the deduced TUD1 protein sequence has high similarity to several sequences in other plant species: 90% identity to 01g042180 protein from *Sorghum bicolor*, 90% to LOC100281502 and 88% to LOC100383857 proteins from *Zea mays*, 60% to AT3G49810 and 56% to AT5G65920 proteins from Arabidopsis (Figure S10). Therefore, the function of TUD1 may be conserved in higher plants.

We examined whether TUD1 possesses a ubiquitination E3 ligase activity as a predicted U-box protein. Ubiquitination activity was observed for the purified GST-TUD1, compared to 2 well-characterised E3 ligases RMA1 and CIP8 proteins [46] (Figure 6D). In addition, tud1-1, tud1-3 and tud1-4 proteins did not possess any apparent E3 ligase activity (Figure 6E), showing that the ubiquination activity of TUD1 is essential for its function.

### TUD1 predominantly localizes to the plasma membrane and physically interacts with D1

**Figure 7:**
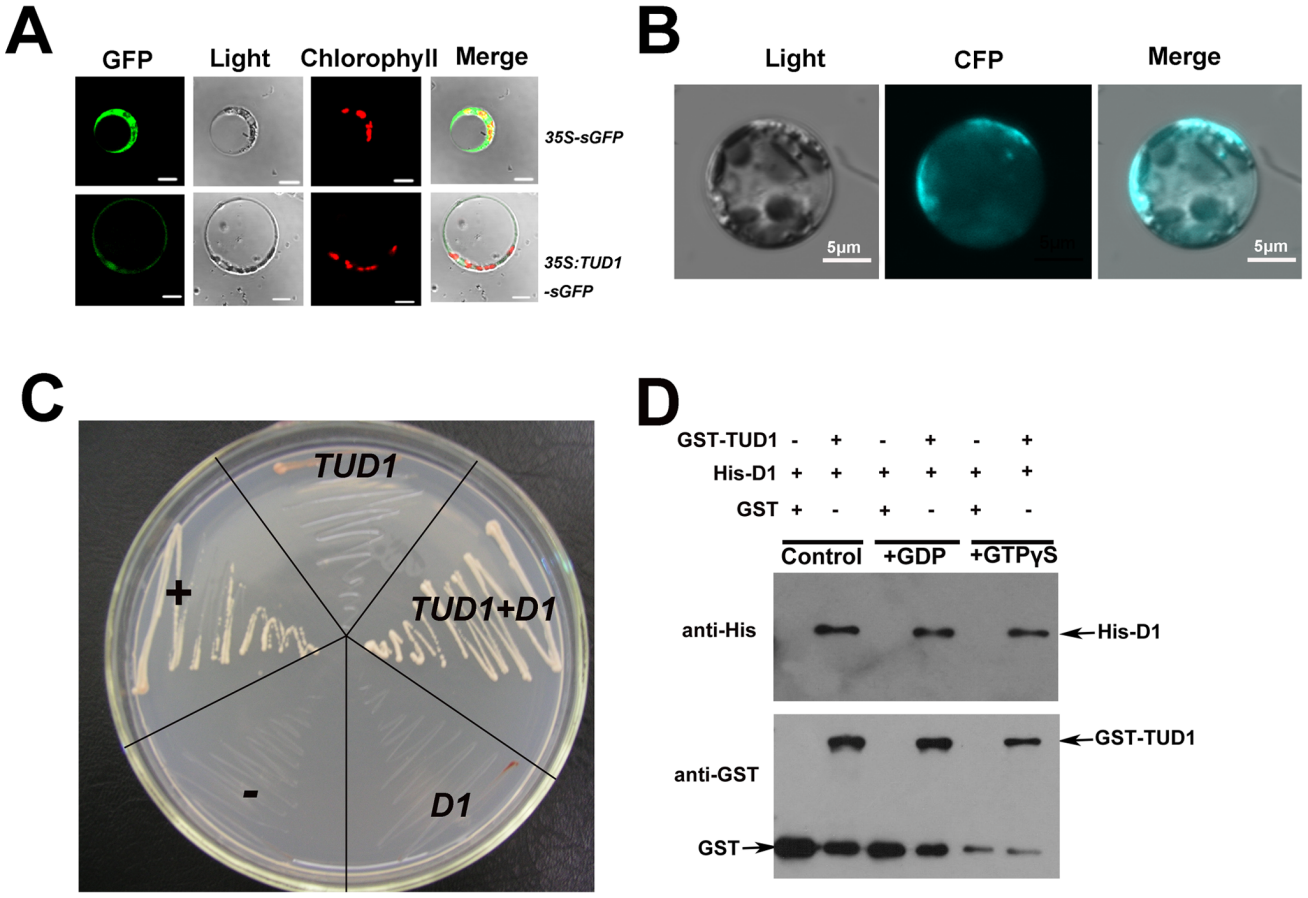
TUD1 is predominantly associated with the plasma membrane and physically interacts with D1. (A) Expression of the TUD1-GFP fusion protein in rice protoplasts. The *35S:sGFP* and *35S:TUD1-sGFP* constructs were transformed into mesophyll protoplasts prepared from rice seedlings and the expression of the introduced genes was viewed after 16 h by confocal microscopy under dark-field or light-field conditions. Bar: 10 µm. (B) BiFC detection of the TUD1-D1 interaction in rice protoplasts. CFP: cyan fluorescence protein. Bar: 5 µm. (C) Interaction between TUD1 and D1 detected by yeast two-hybrid assays. The full-length *D1* and *TUD1* cDNAs were cloned into *pGADT7* and *pGBKT7*, respectively. Yeast AH109 cells were transformed with the vectors indicated and are shown after three days on selective medium. “+” and “−” represent positive and negative control, respectively. *D1* or *TUD1* alone shows weak self-activation. *TUD1*+*D1* show yeast cells transformed with *pGBKT-TUD1* and *pGADT7-D1* together. (D) Pull-down assay for TUD1−D1 interaction. Purified D1 fusion protein was incubated in buffer with either GDP or GTPγS or blank (control) for 2 hours before adding the GST-TUD1 to the binding assay buffer, then precipitated with glutathione-agarose beads. The precipitates were separated by gel electrophoresis and probed with anti-His or anti-GST, respectively.

To investigate the subcellular localization of TUD1, we conducted an *in-vivo* targeting experiment using fusions of TUD1 with synthetic green fluorescent protein (sGFP) as a fluorescent marker in a transient transfection assay. The TUD1::sGFP fusion protein in rice protoplasts was mainly associated with the plasma membrane (Figure 7A), similar to that of D1 [20].

Together with the result that *tud1* is epistatic to *d1*, we wondered whether TUD1 would physically interact with D1. We examined this possibility in three ways. First, we used a biomolecular fluorescence complementation (BiFC) assay [47] to test the interaction between the TUD1 and D1 in rice protoplasts. Cyan fluorescence protein (CFP) fluorescence was reconstituted when the full-length TUD1 and D1 proteins were co-expressed in rice protoplasts (Figure 7B), showing that they physically interact with each other *in vivo*. Second, yeast two-hybrid assays [48] were performed using the D1 or TUD1 and the interaction between TUD1 and D1 was subsequently detected (Figure 7C). Third, we performed a glutathione S-transferase pull-down assay [48] for which we also determined whether TUD1 preferentially interacts with the GTP-bound form of D1. Thus, D1-tagged His and TUD1-tagged GST were expressed and purified from *E. coli* by nickel and glutathione S-transferase columns, respectively. Purified D1 fusion protein was incubated in buffer with either GDP or GTPγS or blank for 2 hours before adding the GST-TUD1 to the binding assay buffer. We subsequently detected that both the GDP- and GTPγS-bound forms of D1 have similar binding ability to GST-TUD1, whereas no binding occurred to GST alone (Figure 7). Based on these results, we concluded that TUD1 physically interacts with D1.

## Discussion

### TUD1 functions together with D1 in the plasma membrane

Heterotrimeric G proteins, consisting of α, β and γ subunits (Gα, Gβ and Gγ), function as signal mediators at the cell plasma membrane in mammals and higher plants [16], [17]. These G protein complexes dissociate into an α subunit and the βγ dimers upon activation of the complex by signal perception in mammals, yeast and higher plants [49].

In rice, previous studies demonstrated that Gα(D1),Gβ, Gγ1, and Gγ2 are not only localized in the plasma membrane, but are also present in a large protein complex (ca. 400 kDa) [19], [20]. As the molecular mass of the αβγ trimer is 100 kDa, the rice 400 kDa G protein complex should contain additional proteins [20]. TUD1 is also localized in the plasma membrane and directly functions with D1 in the plasma membrane. TUD1, therefore, could be associated with the large G protein complex in rice. It is well known that the active Gα forms are free from βγ dimmers, and Gα-GTP monomer and a Gβγ dimmer regulate their own downstream effectors, respectively. However, in our study, pull-down assays showed that TUD1 physically interacts with both the GDP- and GTPγS-bound form of D1, indicating that TUD1 may interact with either inactive or active form of D1. To better understand how D1 and TUD1 interact with each other in vivo, further studies including Co-IP (co-immuoprecipitation) and expressing a constitutively active form of D1 (Q233L) in the *tud1* mutant would be interesting.

Gα subunits and E3 ligases act as integral components of signaling pathways; Gα in the G protein complex and E£ ligase in the ubiquitin-proteasome system (UPS) [17], [23], [27], [28], [32], [33], [34], [35]. However, there was no previous evidence to link Gα with an E3 ligase in any signaling pathway in plants. Now, in our study, we have demonstrated that first link; TUD1 is a functional U-box E3 ligase and directly acts downstream of the Gα subunit D1. Further, *TUD1* and *D1* mutants show impairment in BR responses. Together these results suggest we have uncovered a novel signaling pathway controlling rice growth, whereby a BR signal, mediated by heterotrimeric G protein, is then potentiated into the UPS. It is well-known that a signal perceived by G proteins is generally primary, and so we suggest that the D1-TUD1 interaction could mediate a very early BR response. This proposal is also consistent with the observation of the defective phenotypes in *d1* and *tud1* being at early developmental stages. As yet, we cannot discern whether the BR signal mediated by D1-TUD1 originates from the OsBRI1- mediated BR signaling pathway or other unknown BR receptor(s). It will be interesting to isolate the target(s) of TUD1 to further define this pathway.

### Possible overlaps of the D1-TUD1- and OsBRI1-mediated BR signaling pathways

The well-characterized BRI-mediated BR signaling pathway is conserved among several plant species [1], [2]. Gα also appears to be involved in a BR signaling pathway, but as yet has not been linked to BRI1 [13], [14], [15]. In rice, it is presently regarded as the D1-mediated BR pathway. One possibility is that the D1-mediated BR pathway is parallel to the BRI-mediated BR signaling pathway. The second possibility is that D1 may amplify some BR responses that are initiated by OsBRI1 [14].

In our study, *tud1* mutant was classified as a BR-deficient mutant in the broad sense, with its phenotype more close to *d1* than other reported BR-deficient mutants, such as *d61*, *brd1*, and *d2* [6], [42], [44]. The *tud1* and *d1* mutants have common, characteristic phenotypes of BR-related mutants, but also show short, compact panicles and more specifically decreased length in the vertical direction of grain shape. Histological observation showed that the dwarfism in most of aerial organs of *tud1* was mainly due to a decreased cell proliferation, which is similar to the cause of dwarfism in *d1*. Furthermore, *tud1* is completely epistatic to *d1* and TUD1 functions together with D1 *in vivo*. Based on these results, we conclude that TUD1 is a direct downstream factor of D1 signaling and mediates a G-protein signaling pathway for BR.

Despite TUD1 and D1 showing differences to BRI1, we found that the dwarfism in the second internode and leaf blade of *tud1-2* (due to disorganized cell files and failure of normal cell elongation) is similar to the phenotypes in BR-deficient mutants, such as *d61* and *brd1*. In addition, q-RT-PCR results showed that mutations in TUD1 may lead to an impairment of the OsBRI1-mediated BR pathway transduction. Thus, the D1-TUD1-mediated BR signaling pathway might have an overlapping function with the OsBRI1-mediated BR pathway in the second internode and leaf blade.

In rice, dm-type mutants are commonly identified as BR-deficient mutants [6], [14], [42], [43], [44], but why the second internode is specifically inhibited remains unknown. It is difficult to explain if it is simply due to differential *OsBRI1* expression in different internodes, leading to differences in BR signal strength, through only one OsBRI1-mediated BR pathway. Rather, it suggests there should be additional signal pathway(s) involved in the second internode elongation [6]. Our results support this idea, as we show that the OsBRI1- and D1-TUD1-mediated BR signaling pathways may both have important roles in regulating the second internode elongation in rice. However, there are many questions remaining. For example, how do these two BR signaling pathways function together in the second internode? How are the BR signals perceived and transduced by the D1-TUD1-mediated pathway if not via BRI1? Is the D1-TUD1-mediated BR pathway involved in the signal amplification of some responses that originate from OsBRI1?

### Potential application of the D1-TUD1-mediated signaling pathway in enhancing rice yield

Despite the simple repertoire of G protein signaling elements in plants, multiple signals can be propagated through the G-proteins, to mediate diverse physiological responses [19], [20]. It is well-known that some physiological responses are mainly accounted for by Gα, whereas others are predominantly mediated by Gβγ. In particular, any given G protein component may have multiple differential roles during plant growth and may have different responses to biotic and abiotic stress in a cell-type- or developmental-stage-specific manner [50], [51]. In this context, two important rice yield QTLs, GS3 and DEP1, were recently identified as atypical G protein Gγsubunits [52], [53], [54]. This could serve as a good example for selection and their uses in practical cereal breeding.

In our study, D1 and TUD1 function together not only to promote plant height, panicle development and seed length increase, but also to control a hypersensitive response to infection by avirulent races of a rice blast fungus (our unpublished data). Based on the D1-TUD1-mediated pathway having dual roles in promoting plant growth and resistance to rice blast, it may be feasible to manipulate the components of this pathway to enhance rice yield. Furthermore, similar genes to *TUD1* are found in both Arabidopsis and several cereal species, such as *Sorghum bicolor* and *Zea mays* (Figure S10). As the D1-TUD1-mediated BR signaling pathway is likely conserved across flowering plants, it may provide a way to manipulate the G protein pathway that leads to increased plant productivity.

## Materials and Methods

### Plant materials and growth conditions

The *dwarf1* (*d1-c*), *slender1* (*slr1-l*), *d61-2* mutants were kindly provided by Dr. Chengcai Chu, Da Luo and Makoto Mastsuoka, respectively. Among them, *d1-c* and *slr1-l* were new allelic mutants. The *tud1-1* was derived from tissue culture of an indica cultivar-MH63 (*Oryza sativa* cv. MingHui63), and *tud1-2*, *tud1-3*, and *tud1-4* were isolated from spontaneous mutations of indica cultivars, ZhangYe (ZY), SHuHui163 (SH163), and TeTePu (TTP), respectively. The *tud1-5* was derived from chemical mutagenesis with ethyl methylsulfonate of japonica cultivar Nipponbare.

Double mutants were isolated by phenotype observation and verified by genotyping (the primers used for genotyping are listed in Table S3). For ascertaining the genetic relationship between *tud1-5* and *d1-c*, we successively investigated the segregation ratio of mutations in F_2_ and F_3_ populations of *tud1-5*/*d1-c*. The phenotypic segregation ratio of F_2_ fitted to 9 (normal)∶3 (*d1-c*)∶4 (*tud1-5*). This result was further confirmed by analyzing the F_3_ seeds from homozygous *d1-c* and *tud1-5* plants. F_3_ progeny of *d1-c* plants occasionally segregated the *tud1-5* phenotype in 1∶3 ratio, but F_3_ progeny of *tud1-5* never showed the *d1-c* phenotype (Table S1). Mutants and wild-type rice plants were planted in paddy fields under natural conditions or greenhouse at 30°C (day) and 24°C (night).

### Histological analysis and microscopy observation

Longitudinal sections of the third leaf sheaths and the third internodes were analyzed by light microscopy. Cells of the inner epidermal tissues of lemma were analyzed by scanning electron microscopy. Cell lengths were measured in each organ, both in WT and in *tud1-2* and the total cell number in each organ was estimated. For light microscopy, leaf sheaths and internodes were fixed with formalin: glacial acetic acid: 70% ethanol (1 ∶ 1 ∶ 18) and then dehydrated in a graded ethanol series. Fixed tissues were embedded in paraplast (Sigma), and cut using a microtome into 12 µm thick sections and then applied to glass slides. The sections were counter-stained by 0.005% (w/v) safranin. Tissues were observed under a light microscope (BX51; Olympus, Tokyo, Japan). The inner epidermal cells of lemma were observed by scanning electron microscopy (SEM) (S-3000N; Hitachi, Tokyo, Japan). The samples were photographed under the microscopes and the size of >50 cells in each tissue were measured.

### Real-time RT–PCR

Total RNA was extracted from the third leaf, the uppermost and the second internode using a Invtrogen Extraction kit. Total RNA was treated with RNase-free DNase (Promega; http://www.promega.com) and first strand cDNA was synthesized using SuperScriptII reverse transcriptase (Invtrogene). Real-time PCR was performed using 2×SYBR Green PCR Master Mix (Applied Biosystems) on an Applied Biosystems 7900HT Real-Time PCR System with at least three PCR replicates for each sample. The PCR conditions were 2 min at 50°C, then 10 min at 95°C, followed by 40 cycles of 15 s at 95°C, and 1 min at 60°C.

In addition, methods for GA and BR sensitivity test, map-based cloning, and assays for E3 ubiquitin ligase, subcellular localization, yeast two-hybrid, BiFC and pull-down are provided in Text S1.

## Supporting Information

Figure S1 Relative lengths of each internode of the total culm of *tud1* alleles and their corresponding wild type. The lengths of the upper five internodes of 5 culms were averaged in *tud1* alleles and their corresponding wild type.
(TIF)

Figure S2 Longitudinal sections of the second internode from wild type (WT) (A) and *tud1-2* (B). Bar:100 µm.
(TIF)

Figure S3 Longitudinal sections of the second internode from wild type (WT) (A) and *tud1-1* (B). Bar:100 µm.
(TIF)

Figure S4 Longitudinal observation of the adaxial surface of leaf blade of wild type (WT) and *tud1-2*. (A) and (C):WT; (B) and (D):*tud1-2*. (A) and (B) observed by 10× objective lens. Bar: 2 mm. (C) and (D) observed by 40×objective lens. Bar: 100 µm.
(TIF)

Figure S5 The length of seedling seminal roots of the wild type (WT) and *tud1-2* with or without treatment of 1 µm 6-BA.
(TIF)

Figure S6 The effects of 6-BA on the expression of cytokinin (CK)-related genes in wild type (WT) and *tud1-2*. A: *OsIP4*; B: *OsRR1*; C: *OsHP2*.
(TIF)

Figure S7 Effect of 24-eBL on root elongation in wild-type (WT) and *tud1* seedlings. Seeds of wild type (*TUD1*) and the dwarf mutant (*tud1-2*) were germinated on agar plates in the presence (+) or absence (−) 1 µM of 24-eBL (A). Roots of seedlings were examined 7 days after germination. Bar: 2 cm. The plants were germinated as in (A) with indicated concentrations of 24-eBL.The data presented are the means of the results from a total of five plants (B). Error Bars = SD.
(TIF)

Figure S8 Longitudinal observations of the cleared lamina regions of the wild type (WT) and *tud1-2* treated with (+) or without (−) 100 ng of 24-eBL. Bar: 100 µm.
(TIF)

Figure S9 The effects of 24-eBL on the expression of BR-related genes in wild type (WT), *tud1-2*, *d1-c* and *tud1-2/d1-c* double mutant. A: *BRD1*; B: D2; C:*D61*.
(TIF)

Figure S10 Protein sequence alignment of the U-box domain of Os03g23260 (TUD1) with the domains from its homologs in other plant species. 01g042180 (*Sorghum bicolor*), LOC100281502 and LOC100383857 (*Zea mays*), AT3G49810 and AT5G65920 (Arabidopsis). Red box indicates U-box domain.
(TIF)

Table S1 Phenotypic Variations in the F_2_ or F_3_ Generations of *tud1* Crossed with *d1*.
(DOC)

Table S2 Cell Length and Cell Number in the Third Leaf Sheath the Third Internode and the Lemma in WT and *tud1-2*.
(DOC)

Table S3 The Primers Used in This Study for Genotyping the Mutants.
(DOC)

Table S4 Primers for Map-based Cloning *TUD1*.
(DOC)

Table S5 Primers for q-PCR.
(DOC)

Text S1 Supplemental Methods.
(DOCX)
