# Supplementary figures and images for: The U-Box E3 Ubiquitin Ligase TUD1 Functions with a Heterotrimeric G α Subunit to Regulate Brassinosteroid-Mediated Growth in Rice
Source: PLoS Genet. 2013 Mar 14;9(3):e1003391. doi: 10.1371/journal.pgen.1003391 (PMC3597501; doi:10.1371/journal.pgen.1003391)

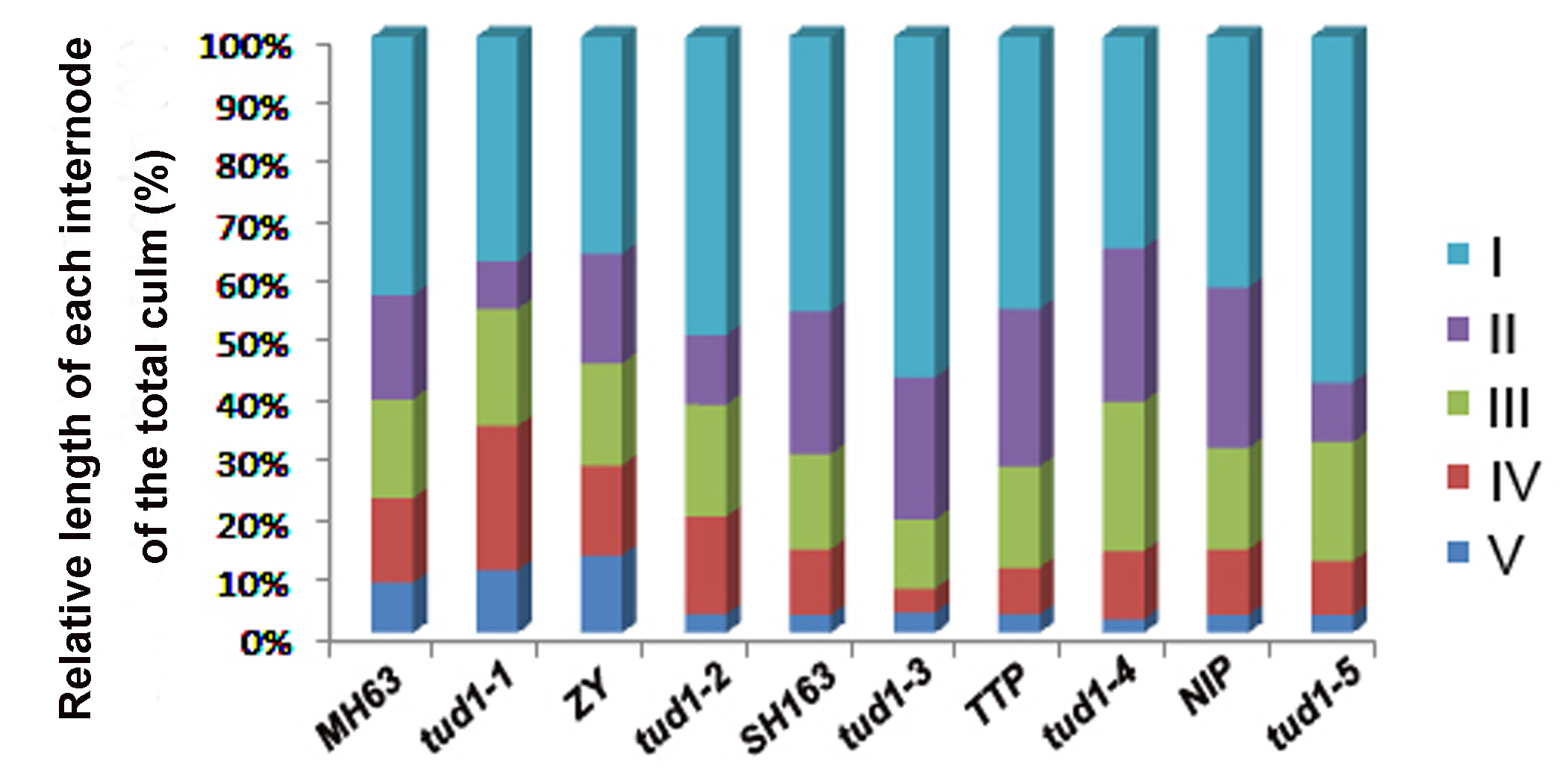

Supplement: Figure S1 — Relative lengths of each internode of the total culm of tud1 alleles and their corresponding wild type. The lengths of the upper five internodes of 5 culms were averaged in tud1 alleles and their corresponding wild type. (TIF) [file pgen.1003391.s001.tif]

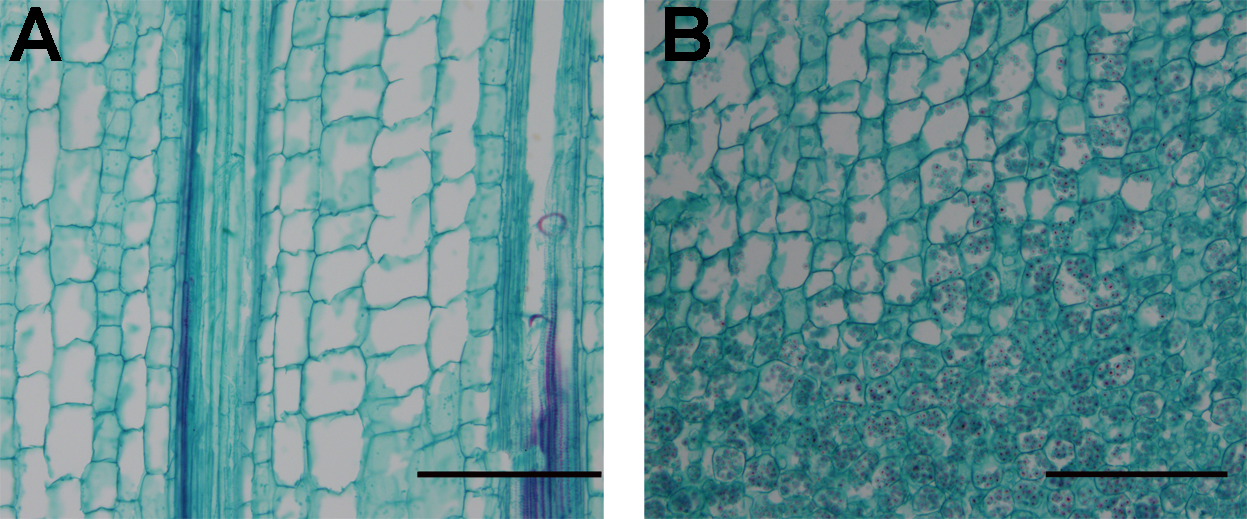

Supplement: Figure S2 — Longitudinal sections of the second internode from wild type (WT) (A) and tud1-2 (B). Bar:100 µm. (TIF) [file pgen.1003391.s002.tif]

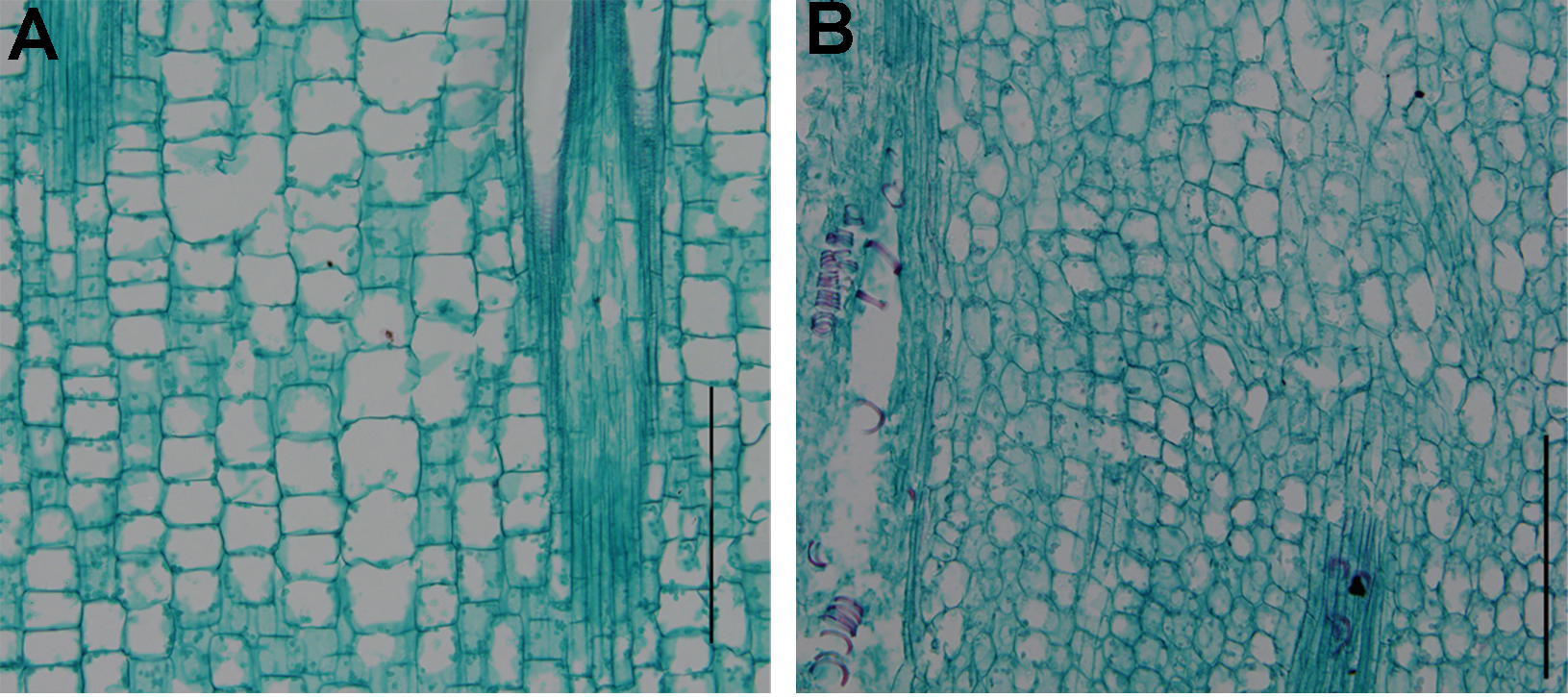

Supplement: Figure S3 — Longitudinal sections of the second internode from wild type (WT) (A) and tud1-1 (B). Bar:100 µm. (TIF) [file pgen.1003391.s003.tif]

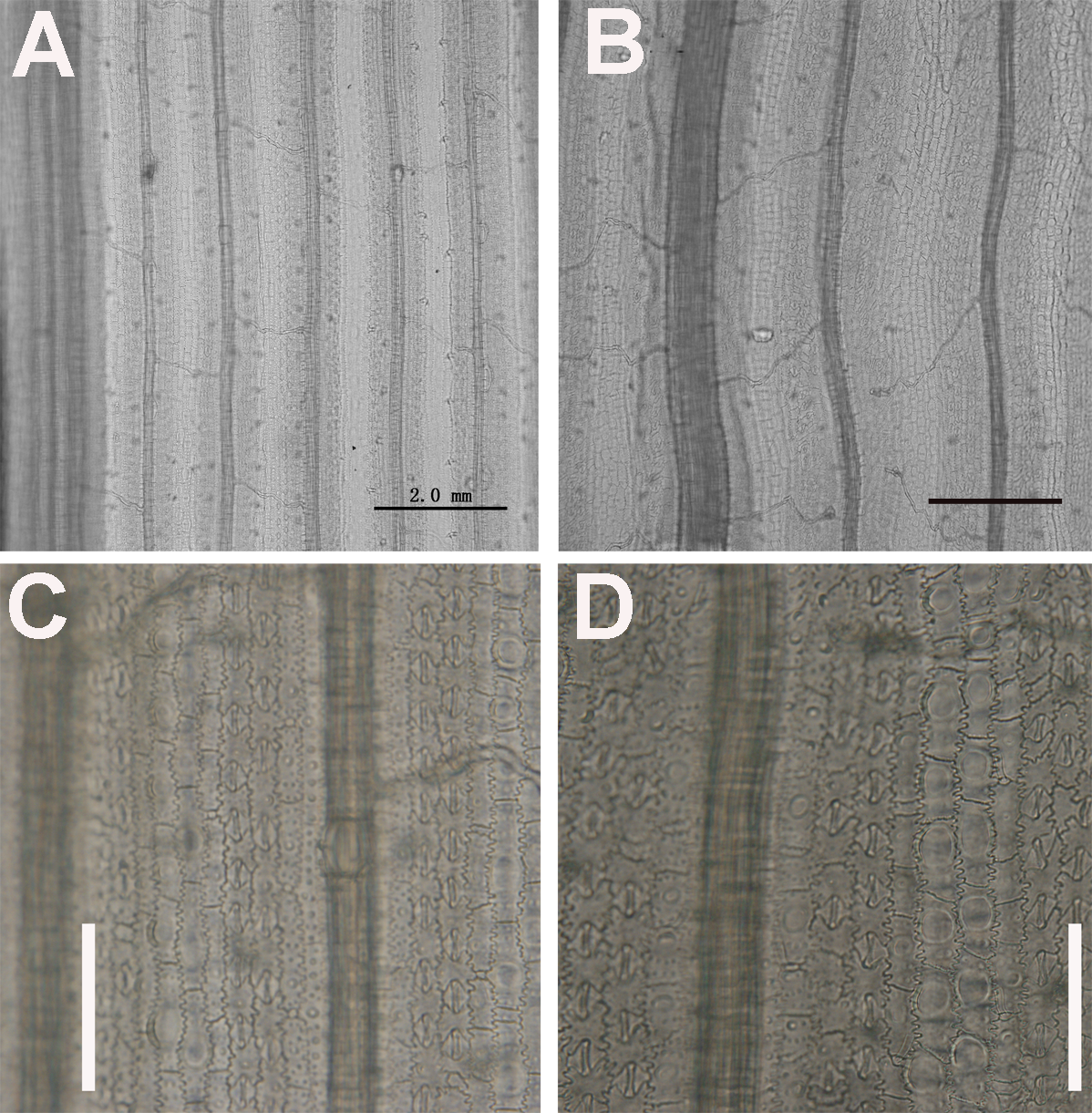

Supplement: Figure S4 — Longitudinal observation of the adaxial surface of leaf blade of wild type (WT) and tud1-2. (A) and (C):WT; (B) and (D):tud1-2. (A) and (B) observed by 10× objective lens. Bar: 2 mm. (C) and (D) observed by 40×objective lens. Bar: 100 µm. (TIF) [file pgen.1003391.s004.tif]

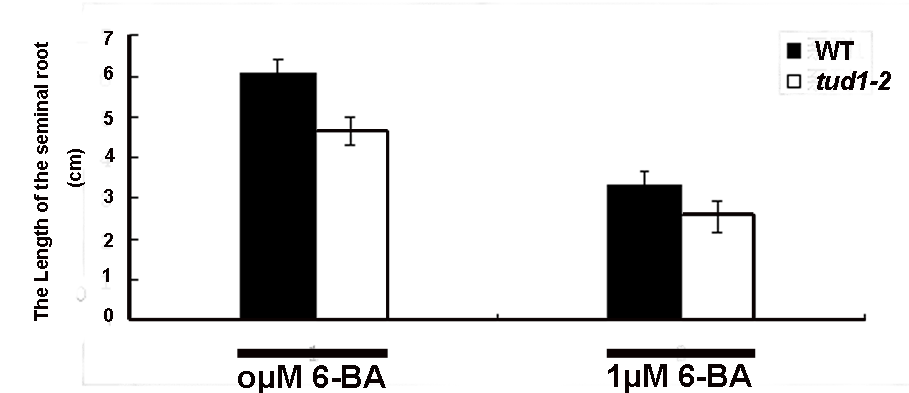

Supplement: Figure S5 — The length of seedling seminal roots of the wild type (WT) and tud1-2 with or without treatment of 1 µm 6-BA. (TIF) [file pgen.1003391.s005.tif]

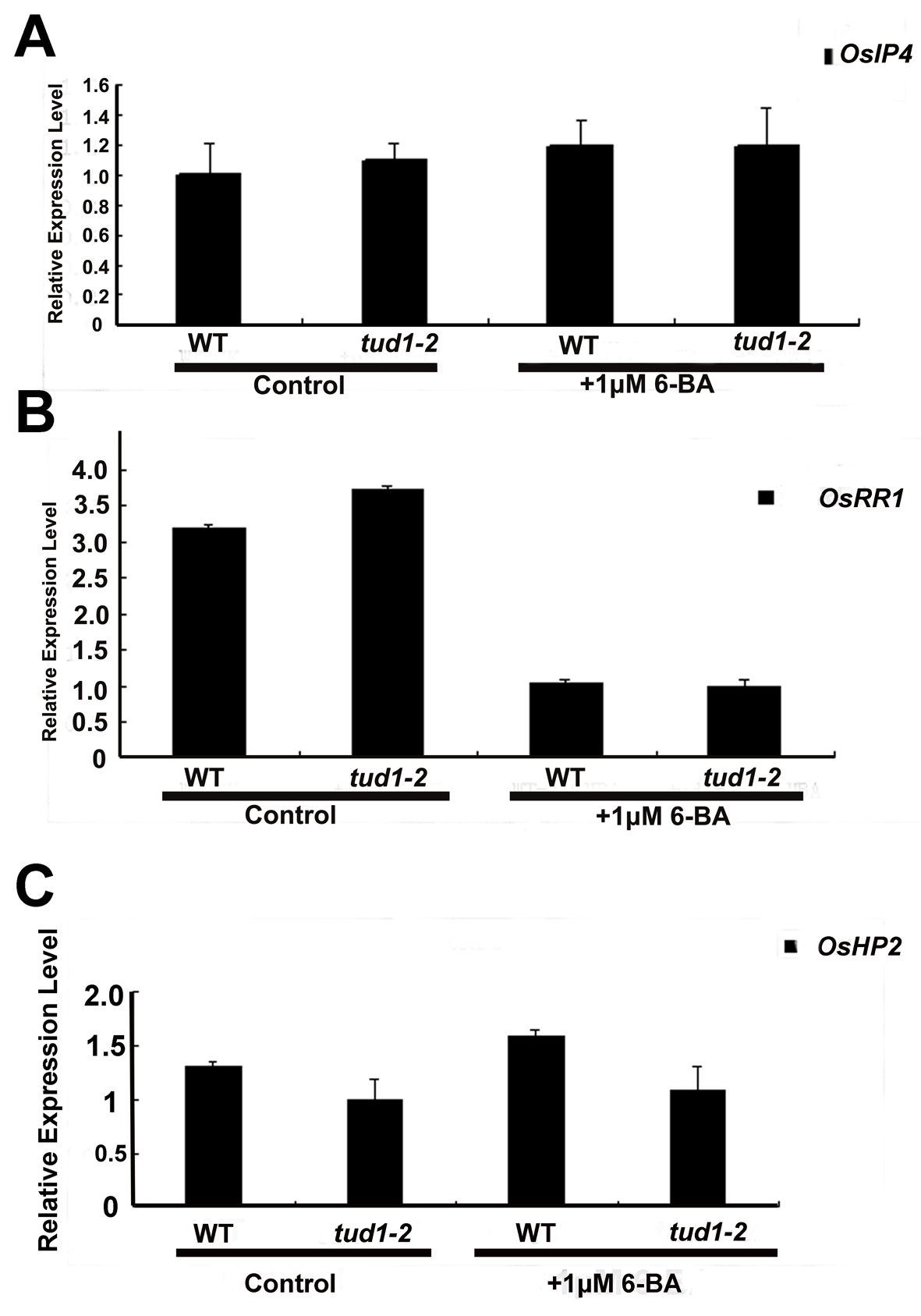

Supplement: Figure S6 — The effects of 6-BA on the expression of cytokinin (CK)-related genes in wild type (WT) and tud1-2. A: OsIP4; B: OsRR1; C: OsHP2. (TIF) [file pgen.1003391.s006.tif]

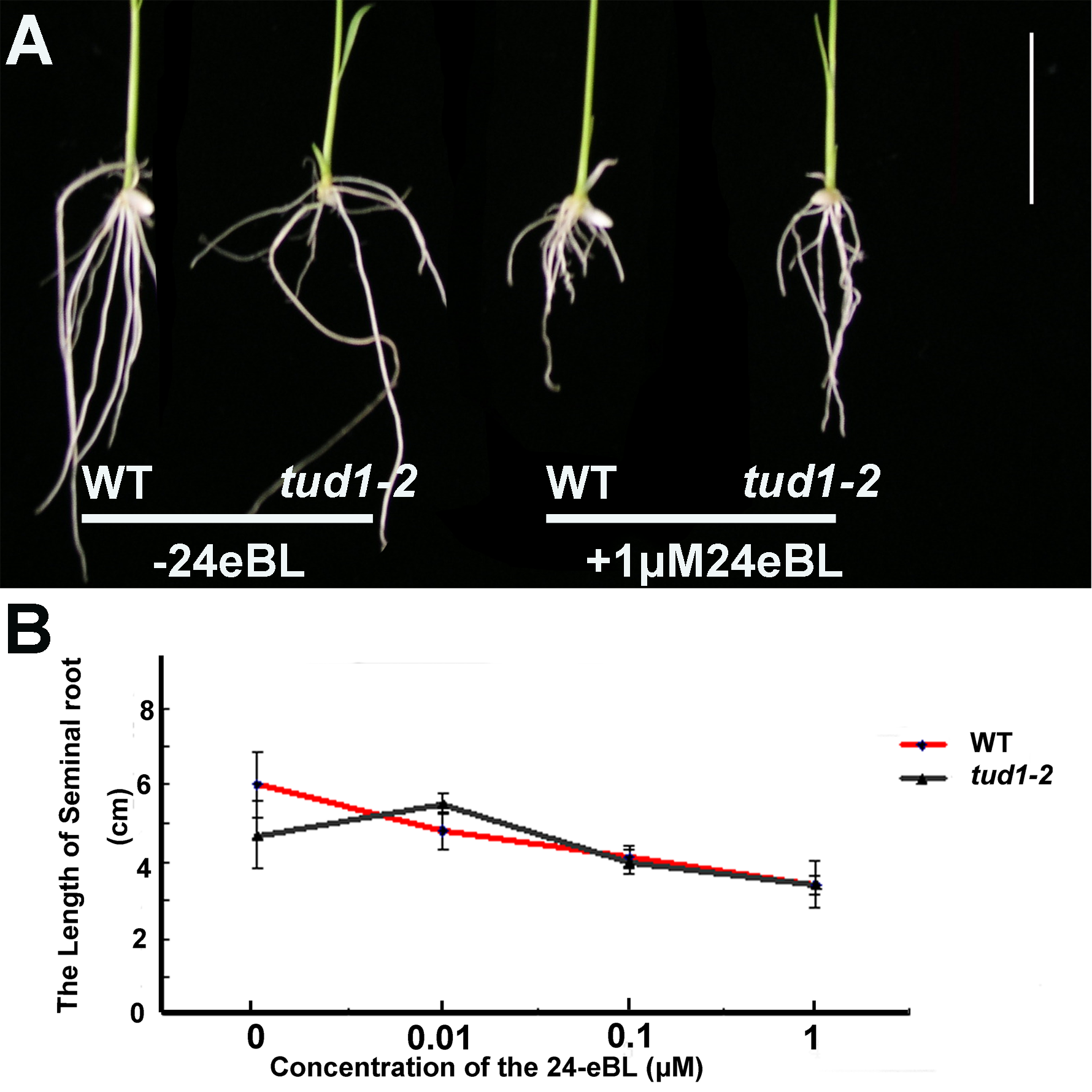

Supplement: Figure S7 — Effect of 24-eBL on root elongation in wild-type (WT) and tud1 seedlings. Seeds of wild type (TUD1) and the dwarf mutant (tud1-2) were germinated on agar plates in the presence (+) or absence (−) 1 µM of 24-eBL (A). Roots of seedlings were examined 7 days after germination. Bar: 2 cm. The plants were germinated as in (A) with indicated concentrations of 24-eBL.The data presented are the means of the results from a total of five plants (B). Error Bars = SD. (TIF) [file pgen.1003391.s007.tif]

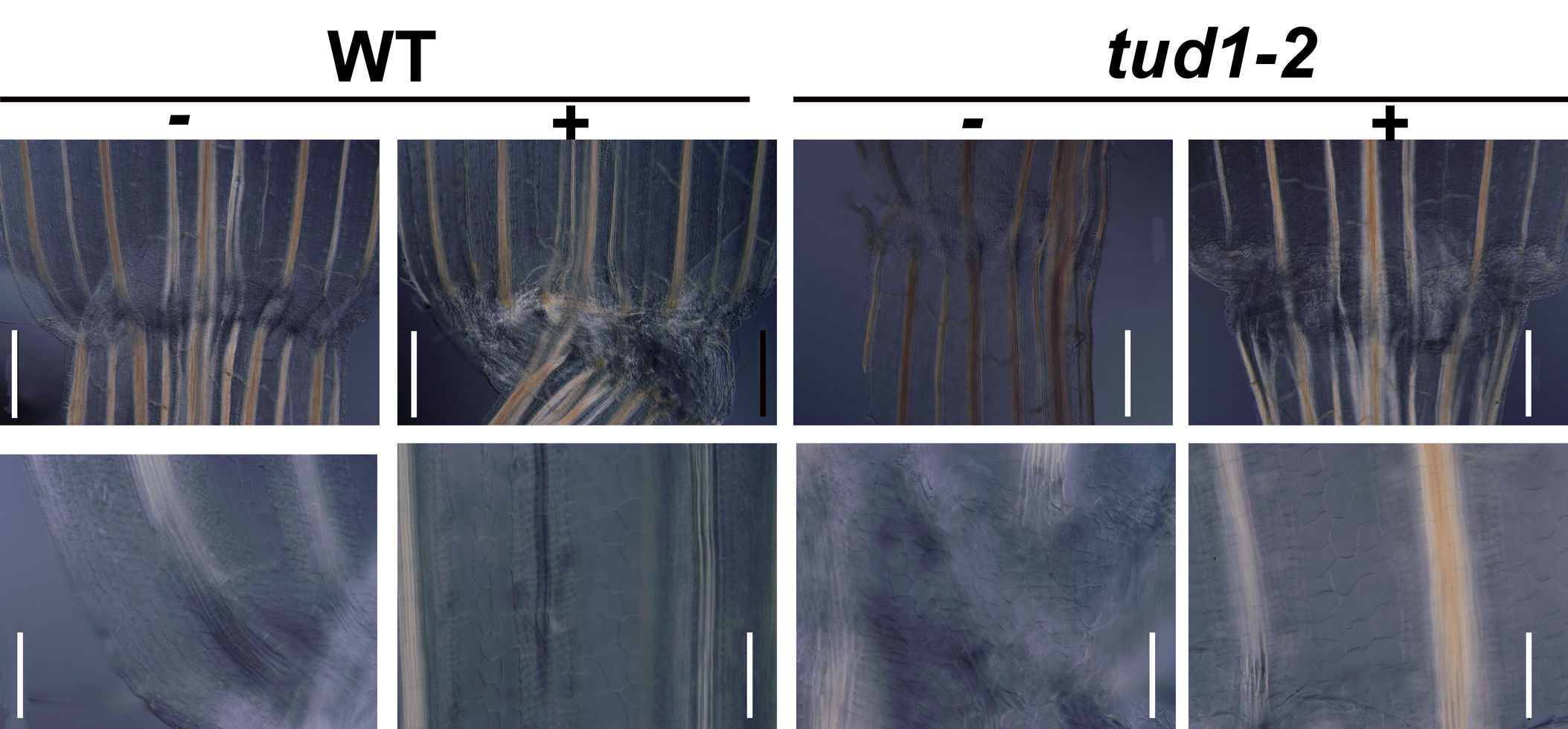

Supplement: Figure S8 — Longitudinal observations of the cleared lamina regions of the wild type (WT) and tud1-2 treated with (+) or without (−) 100 ng of 24-eBL. Bar: 100 µm. (TIF) [file pgen.1003391.s008.tif]

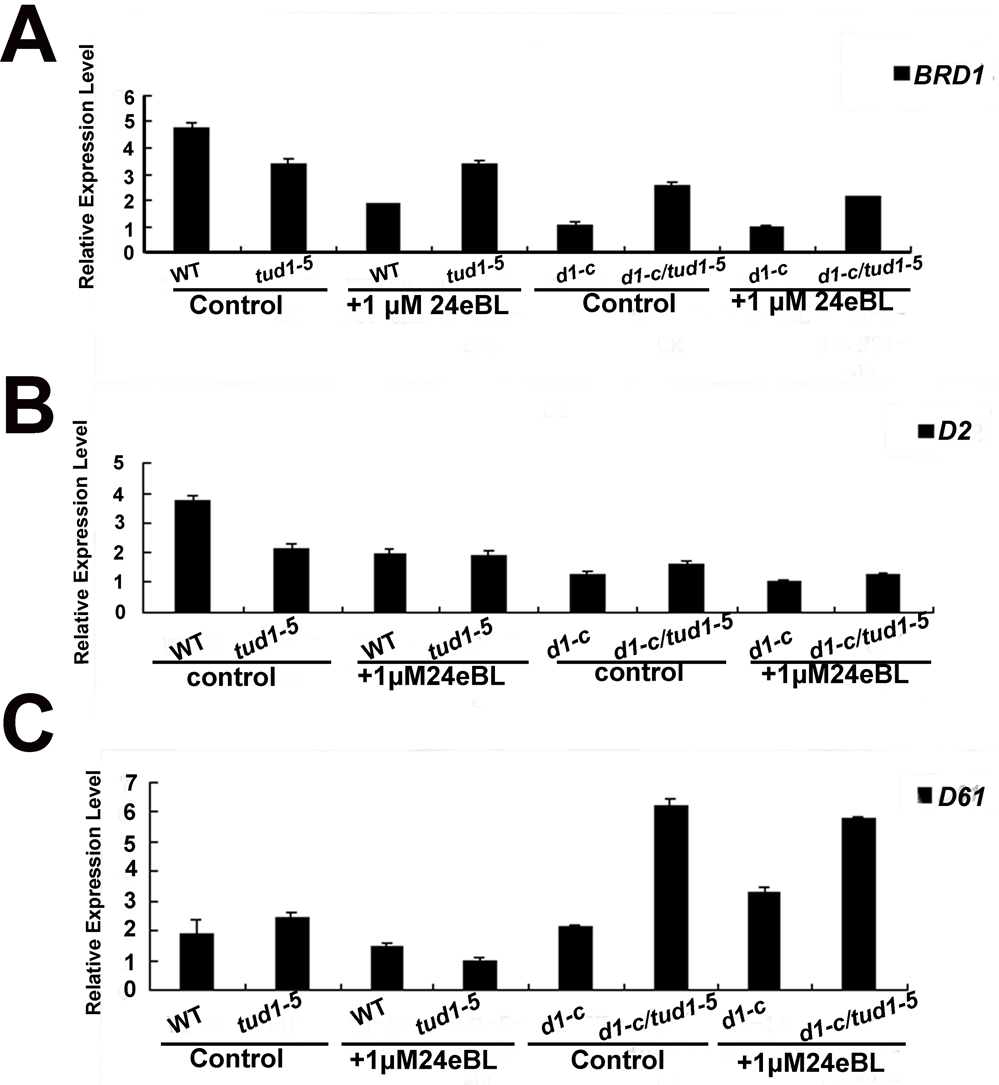

Supplement: Figure S9 — The effects of 24-eBL on the expression of BR-related genes in wild type (WT), tud1-2, d1-c and tud1-2/d1-c double mutant. A: BRD1; B: D2; C:D61. (TIF) [file pgen.1003391.s009.tif]

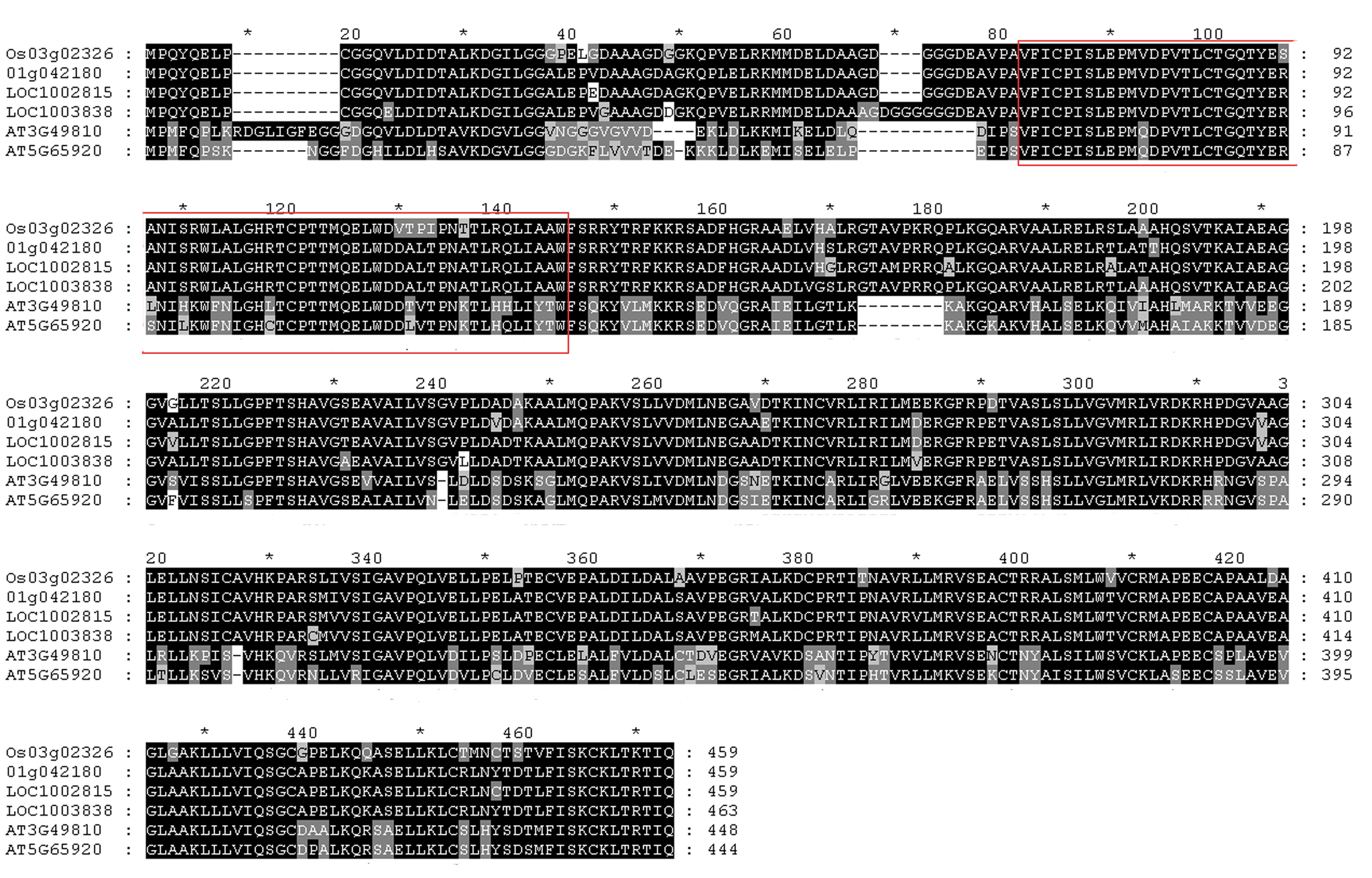

Supplement: Figure S10 — Protein sequence alignment of the U-box domain of Os03g23260 (TUD1) with the domains from its homologs in other plant species. 01g042180 (Sorghum bicolor), LOC100281502 and LOC100383857 (Zea mays), AT3G49810 and AT5G65920 (Arabidopsis). Red box indicates U-box domain. (TIF) [file pgen.1003391.s010.tif]
