# Supplementary material for: The U-Box E3 Ubiquitin Ligase TUD1 Functions with a Heterotrimeric G α Subunit to Regulate Brassinosteroid-Mediated Growth in Rice
Source: PLoS Genet. 2013 Mar 14;9(3):e1003391. doi: 10.1371/journal.pgen.1003391 (PMC3597501; doi:10.1371/journal.pgen.1003391)
Supplement: Table S1 — Phenotypic Variations in the F2 or F3 Generations of tud1 Crossed with d1. (DOC) [file pgen.1003391.s011.doc]

Table S1． Phenotypic Variations in the F_2_ or F_3_ Generations of *tud1-5* Crossed with *d1-c*

| Genotype | Phenotype of F_2_ or F_3_ Progeny | | | | χ^2^（9:3:4,  3:1 or 1:0) | *P* |
| --- | --- | --- | --- | --- | --- | --- |
|  | Normal | *d1-like* | *tud1-like* | Total |  |  |
| *+tud1-5+d1-c* | 43 | 12 | 20 | 75 | 0.40(9:3:4) | >0.01 |
| *d1-cd1-c+tud1-5* |  | 16 | 9 | 25 | 0.013(3:1) | >0.01 |
| *tud1-5tud1-5+d1-c* |  | 0 | 15 | 15 | 0(1:0) | >0.01 |
