# Supplementary material for: The U-Box E3 Ubiquitin Ligase TUD1 Functions with a Heterotrimeric G α Subunit to Regulate Brassinosteroid-Mediated Growth in Rice
Source: PLoS Genet. 2013 Mar 14;9(3):e1003391. doi: 10.1371/journal.pgen.1003391 (PMC3597501; doi:10.1371/journal.pgen.1003391)
Supplement: Table S2 — Cell Length and Cell Number in the Third Leaf Sheath the Third Internode and the Lemma in WT and tud1-2. (DOC) [file pgen.1003391.s012.doc]

Table S2. Cell Length and Cell Number in the Third Leaf Sheath the Third Internode and the Lemma in WT and *tud1-2*

^a^Values are averages of the five plants (means±SD)

^b^Average cell length in an 8mm portion of the central area of the third leaf sheath (mean±SD, n=306). Significant differences were not detected between WT and *tud1-2*

^c^Average cell length in a 5mm portion of the central area of the third internode (mean±SD,n=67). Significant differences were detected between WT and *tud1-2* (*p<0.005 ,*t-test)

^d^Average cell length in 1mm portion of the central area of the lemma just heading(mean±SD,n=60). Significant differences were detected between WT and *tud1-2* (*p<0.005 ,*t-test)

|  | **Leaf sheath** | **Internode** | **Lemma** |
| --- | --- | --- | --- |
| Organ length^a^(cm) |  |  |  |
| WT | 7.26±0.23 | 12.7±0.6 | 1.1±0.04 |
| *tud1-2* | 3.12±0.08 | 4.8±0.3 | 0.79±0.01 |
| *tud1-2*/WT ratio(%) | 0.43 | 0.38 | 0.72 |
| Cell length(μm) |  |  |  |
| WT | 160.3±33.6^b^ | 31.4±9.17^c^ | 89±23.2^d^ |
| *tud1-2* | 161.1±36.8^b^ | 36.5±10.14^c^ | 99±24.9^d^ |
| *tud1-2*/WT ratio(%) | 100.5 | 116.2 | 111.2 |
| Deduced cell number |  |  |  |
| WT | 453 | 4044 | 124 |
| *tud1-2* | 194 | 1315 | 80 |
| *tud1-2*/WT ratio(%) | 42.8 | 32.5 | 64.5 |
