# Supplementary material for: The U-Box E3 Ubiquitin Ligase TUD1 Functions with a Heterotrimeric G α Subunit to Regulate Brassinosteroid-Mediated Growth in Rice
Source: PLoS Genet. 2013 Mar 14;9(3):e1003391. doi: 10.1371/journal.pgen.1003391 (PMC3597501; doi:10.1371/journal.pgen.1003391)
Supplement: Table S3 — The Primers Used in This Study for Genotyping the Mutants. (DOC) [file pgen.1003391.s013.doc]

Table S3. The Primers Used in This Study for Genotyping the Mutants

| Mutant | Mutation site | Forward primer(5’-3’) | Reverse Primer(5’-3’) |
| --- | --- | --- | --- |
| *d1-c* | 341-550(210bp) deletion in CDS | ATGGGCTCATCCTGTAGCAG | TCAAGTTCCTTCCCTGGAGC |
| *slr1-l* | 737T-C  substitution | ATGAAGCGCGAGTACCAAGAAG | GGCAGGACTCGTAGAAGTGGG |
| *eui1-d* | 814-825(12bp)  deletion in CDS | GGGCTTGCTTTGGGAGTG | CCGCTGTTCTCGATGATGG |
| *d61-2* | 1471G-A  substitution | CCAATCCCTTCATGGCTTGG | GCTCGGCATCCGACTGAGG |
