# Supplementary material for: The U-Box E3 Ubiquitin Ligase TUD1 Functions with a Heterotrimeric G α Subunit to Regulate Brassinosteroid-Mediated Growth in Rice
Source: PLoS Genet. 2013 Mar 14;9(3):e1003391. doi: 10.1371/journal.pgen.1003391 (PMC3597501; doi:10.1371/journal.pgen.1003391)
Supplement: Table S4 — Primers for Map-based Cloning TUD1. (DOC) [file pgen.1003391.s014.doc]

Table S4: The Primers for q-PCR

| Markers | Forward Primers (5'-3') | Reverse Primer (5'-3') |
| --- | --- | --- |
| *OsActin* | 5'-TCCATCTTGGCATCTCTCAG-3' | 5'-GTACCCGCATCGGCATCTG-3' |
| *BRD1* | 5'-GGAGAAGAACDTGGAATCAC-3' | 5'-GTAATCTTGAACGCGGATATG-3' |
| *D2* | 5'-TTCAACCCATGGAGGTGGAA-3' | 5'-GCACGGTGGGGAAGTTGACGA-3' |
| *OsDWARF4* | 5'-AGTCGCGTGCTGCCATTCTCGGAGTAATAG-3' | 5'-AGCAAGCTCAGCAAGAGGTCCAGGATTTGC-3' |
| *D61* | 5'-CTCGGCAGCGTCGAGGTGC-3' | 5'-AGGAATTGTTGCTGAGCTTC-3' |
| *OsIPT4* | 5' -AGCTGAGCTAGCGATCAACAC-3 ′ | 5' -TGTACGCCTGCATGGTGA-3 |
| *OsHP2* | 5'-GTTTGGTGGACGAGCAGTTC-3' | 5'-CTGCTGCCTTTGAGCTGATG-3' |
| *OsRR1* | 5'-AGGATCAGCAGATGCATGAATG-3' | 5'-GAGACGCTGTACGTCCTTGCTT-3' |
