# Supplementary material for: The U-Box E3 Ubiquitin Ligase TUD1 Functions with a Heterotrimeric G α Subunit to Regulate Brassinosteroid-Mediated Growth in Rice
Source: PLoS Genet. 2013 Mar 14;9(3):e1003391. doi: 10.1371/journal.pgen.1003391 (PMC3597501; doi:10.1371/journal.pgen.1003391)
Supplement: Table S5 — Primers for q-PCR. (DOC) [file pgen.1003391.s015.doc]

*Table S5. Primers for Map-based Cloning* TUD1

| Markers | Forward Primer(5’-3’) | Reverse Primer(5’-3’) |
| --- | --- | --- |
| s119341 | GGACATCGGTACTACCATAGTCA | CTCTCGCAATGTCGATCCA |
| s155769 | GAACCGGCAAATGAACATAAG | CGGCTAATATGGTGATATGGAC |
| P1 | AAGCTACGGTTGCCTCCTC | GTTGGATGGGCAGAGGCT |
| P2 | GGTAGCGTCATGGTGTGC | GAACATAAGCCTATTATCACGA |
| P3 | CAGTGAGATCCACATGCCA | CAGTAGGACCCATATGTAACACA |
| P4 | AGCGGCAACGGCATAACAC | CCGCAGCCCCTCCTTCCTC |
| P5 | CCATGTTCTCTCCAGTTCCC | TGCGGGTAATAGAAGAGGAA |
| s99245 | GGGAATAGCAATCAACTGAAA | GAAGTTGTACAGCCTGTCCG |
| s32861 | CAGTGTAAGTCAGACATTTCCG | CTGGACTGGTAATGGTTCTAGG |
