# Supplementary material for: The U-Box E3 Ubiquitin Ligase TUD1 Functions with a Heterotrimeric G α Subunit to Regulate Brassinosteroid-Mediated Growth in Rice
Source: PLoS Genet. 2013 Mar 14;9(3):e1003391. doi: 10.1371/journal.pgen.1003391 (PMC3597501; doi:10.1371/journal.pgen.1003391)
Supplement: Text S1 — Supplemental Methods. (DOCX) [file pgen.1003391.s016.docx]

**Title: A U-Box E3 Ubiquitin Ligase TUD1 Functions Together with Heterotrimeric G α Subunit to Regulate Brassinosteroid-Mediated Growth in Rice**

**Running head: A U-Box E3 Ubiquitin Ligase in G -protein Signaling**

**Xinming Hu^1,2^, Qian Qian^2^, Ting Xu^1,3^, Yu’e Zhang^1^, Guojun Dong^2^** ,**Ting Gao^4^**,**Qi Xie^4^ and Yongbiao Xue^1,5^**

^1^State Key Laboratory of Molecular Developmental Biology, Chinese Academy of Sciences and National Center for Plant Gene Research, Beijing 100101, China

^2^National Rice Research Institute, Chinese Academy of Agricultural Sciences, Hangzhou 310006, China

^3^Graduate University, Chinese Academy of Sciences, Beijing 100190, China

^4^State Key Laboratory of Plant Genomics, Institute of Genetics and Developmental Biology, Chinese Academy of Sciences and National Center for Plant Gene Research, Beijing 100101, China

^5^To whom correspondence should be addressed. E-mail: ybxue@genetics.ac.cn

**Supplemental Methods**

**GA and BR Sensitivity Tests**

For GA sensitivity tests, the second leaf sheath elongation assay was carried out as described previously (21). Lamina joint inclination assay were performed as described previously with slightly modified conditions (13,14). Imbibed seeds were sown on 0.6% agar medium and grown in an incubator room in continuous light at 30°C until the third leaves began to emerge (4 days). Then, various amounts of 24-epiBL (Sigma) were applied to the lamina regions between the second leaf and leaf sheath. After 3 days, the bending angles of the lamina joint were measured. For primary root response to 24-eBL test, coleoptile and mesocotyl elongation in complete darkness assay was followed as previously reported (6).

**Map-Based Cloning and Complementary Test**

Map-based cloning was conducted using a mapping population made by crosses between the *tud1-2* and *japonica* cultivar-Nipponbare. The *tud1-1* mutation was initially mapped on chromosome 3. Using more than 1000 mutant individuals from the mapping population, we screened recombinants between two genetic markers that reside 18 kb or so apart on the physical map and identified five recombinants between these markers (Table S5). This region corresponded to BAC clones AC103891. Mutations in *tud1* were detected by PCR amplification and sequencing of genes predicted within this region.

For the complementation test, a DNA fragment ~6.4kb in size, including the entire sequence of putative *TUD1* gene was introduced into *tud1-2* by the *Agrobacterium tumefaciens*–mediated transformation method (45).

**E3 Ubiquitin Ligase Activity Assay**

The entire *TUD1* open reading frame (1380bp) and *tud1* mutant sequences containing mutations in open reading frame were cloned into *pGEX6P-1* and expressed in *Escherichia coli*. The fusion proteins were prepared according to manufacturer’s instruction. For the E3 ubiquitin ligase activity assay of fusion protein, crude extracts containing recombinant wheat (*Triticum aestivum*) E1 (GI: 136632), human E2 (UBCh5b; 40 ng), purified E3 (1 mg) fused with the GST tag and purified Arabidopsis ubiquitin (UBQ14, At4g02890; 2 μg) fused with the His tag, were used for the assay. *In vitro* E3 ligase assays were performed as described (5). Proteins after reaction were separated by SDS-PAGE, blotted, probed by HisDetector nickel–nitrilotriacetic acid agarose conjugated to horseradish peroxidase (Kirkegaard & Perry Laboratories) for the detection of His-tagged ubiquitin (antiserum; NewEngland Biolabs), and visualized using chemiluminescence as instructed by the manufacturer (ECL; Amersham Pharmacia).

**Subcellular Localization of TUD1**

A full- length cDNA of *TUD1* (CDS) was amplified by PCR using high-fidelity TAQ enzyme (Takara) and primers containing *Bgl*Ⅱand *Sal*I site*s* (*5＇-CCagatctATGCCGCAGTACCAGG AGC-3＇*and *5＇-CCgtcgacGCTTACGAAGACAATCCAG-3＇*)*.*The PCR products was fully sequenced and digested by *Bgl*Ⅱ *and Sal*I*,* then ligated into *pBI221-GFP* transient expression vector which was digested by *Bam*HI and *Sal*I. The resulting *TUD-GFP* fusion constructs driven by the *CaMV35S* promoter were delivered into rice mesophyll protoplasts using a polyethylene glycol (PEG)-calcium-mediated method (6) followed by 12-24 h incubation to allow transient expression. Chlorophyll autofluorescence was used as a chloroplast marker. Expression of these fusion constructs was monitored using a confocal microscope (LSM 510 META, Carl Zeiss, Germany).

**Yeast Two-Hybrid Assay**

To test the interaction between TUD1 and D1, the full-length cDNA of *TUD1* and *D1* were amplified by primer pairs with adaptors for *Eco*RI-*SalI* (*5’-CCGgaattcATGCTGT GGGTGGTGT GTAGG-3’* and *5’-CCgtcgacCTGGATTGTCTTCGTAAGCTTGC -3’*), and *Bam*HI-*Xho*I (*5’-GGggatccATGGGCTCATCCTGTAGCAG-3’*and *5’-CCGctcgagTCAAGTTCCT TCCCTGGAG C-3’*) digestion sites and cloned into *pGBK-T7* and pGAD-T7 (Clontech), respectively. The BD and AD vectors were cotransformed into AH109 and grown on -Leu/-Trp medium containing 2% agar at 30°C for 4 to 5 d. The clones were further grown on -Leu/-Trp/-His/-Ade medium containing 2% agar at 30°C for 3 to 4d to test interaction. For liquid ß-galactosidase assay, yeast clones grown for 48 h at 30°C were transferred onto filter paper, and the clones were lysed in liquid nitrogen for 1 min, and then 5 mL of Z buffer (60 mM Na_2_HPO4,40mMNaH_2_PO4, 10mM KCl, and 1mM MgSO4, pH 7.0) containing ß-mercaptoethanol (27 µL/10 mL) was added, incubated at 30°C (for <8 h) and checked periodically for the appearance of blue color (7).

**BiFC Assays**

BiFC assays were performed as described previously (6). For generation of BiFC vectors, the CDS of *TUD1* was amplified with primer pairs and cloned at *Sal*I-*Kpn*I sites in *pSCYCE*. The CDS of *D1* was cloned into *Sal*I-*Kpn*I sites of *pSCYCE* (R). Rice mesophyll protoplasts were prepared and transformed with combinations of constructs and imaged for fluorescence of CFP as mentioned above.

**Pull-Down Assay**

Recombinant fusion proteins His-D1 and GST-TUD1 were purified from BL21 (DE3) cells using affinity chromatography according to manufacturer’s instruction (Novagen). His-D1 was loaded with GDP or GTPγS before adding GST-TUD1 to test the specific binding of D1 to TUD1 in buffer (50 mM Tris-HCl, pH 7.5, 50 mM NaCl, 2 mMDTT, 5 mM MgSO4, protease inhibitor cocktail for bacterial cell extracts[Sigma-Aldrich], and 100 mM GDP or GTPγS. The samples were incubated at 4℃ for 2 h in the presence of glutathione-agarose, washed five times with the lysis buffer (20 mM Tris-HCl, pH 8.0 200 mM NaCl, 11 mM EDTA pH 8.0, 0.5% Nonidet P-40, 2 μg/μl aprotinin, 1 μg/μl leupeptin, 0.7 μg/ml pepstatin and 25 μg/ml phenylmethylsulfonyl fluoride (PMSF)) and eluted with buffer 20 mM reduced glutathione in 50 mM Tris-HCl (pH 8.0). Proteins were separated by SDS-PAGE and immunoblotted with anti-GST and anti-His (Sigma-Aldrich), respectively.
